# Oxy-imino saccharidic derivatives as a new structural class of aldose reductase inhibitors endowed with anti-oxidant activity

**DOI:** 10.1080/14756366.2020.1763331

**Published:** 2020-05-12

**Authors:** Felicia D'Andrea, Stefania Sartini, Ilaria Piano, Matteo Franceschi, Luca Quattrini, Lorenzo Guazzelli, Lidia Ciccone, Elisabetta Orlandini, Claudia Gargini, Concettina La Motta, Susanna Nencetti

**Affiliations:** a Department of Pharmacy, University of Pisa, Pisa, Italy; b Department of Earth Sciences, University of Pisa, Pisa, Italy; c Research Center “E. Piaggio”, University of Pisa, Pisa, Italy

**Keywords:** Diabetes, aldose reductase inhibitors, aldohexos-5-uloses, oxy-imino saccharidic derivatives, azapyranose

## Abstract

Aldose reductase is a key enzyme in the development of long term diabetic complications and its inhibition represents a viable therapeutic solution for people affected by these pathologies. Therefore, the search for effective aldose reductase inhibitors is a timely and pressing challenge. Herein we describe the access to a novel class of oxyimino derivatives, obtained by reaction of a 1,5-dicarbonyl substrate with *O*-(arylmethyl)hydroxylamines. The synthesised compounds proved to be active against the target enzyme. The best performing inhibitor, compound (*Z*)-**8,** proved also to reduce both cell death and the apoptotic process when tested in an *in vitro* model of diabetic retinopathy made of photoreceptor-like 661w cell line exposed to high-glucose medium, counteracting oxidative stress triggered by hyperglycaemic conditions.

## 1. Introduction

Diabetes mellitus (DM) is a worldwide health problem affecting approximately 415 millions of people and is expected that this number will grow rapidly in the next two decades^1^. The increase of diabetic patients is associated to lifestyle changes, obesity, physical inactivity, and possibly a genetic predisposition^2–4^. Patients with diabetes mellitus suffer from long-term macrovascular and microvascular complications, leading over time to cardiovascular pathologies, neuropathy, nephropathy, some form of retinopathy, glaucoma and cataracts, which severely limit their life quality.

**Figure 1.**
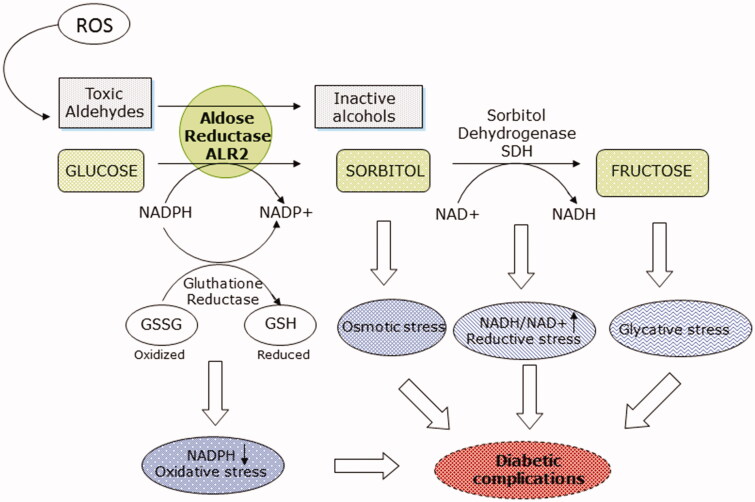
Role of aldose reductase in diabetic complications.

Aldose reductase (ALR2, EC 1.1.1.21) is a target protein associated with diabetic complication and is the key enzyme of the polyol pathway^5^. The enzyme catalyses the NADPH-dependent reduction of glucose to sorbitol which is subsequently oxidised to fructose by sorbitol dehydrogenase (SDH) through a NAD^+^ dependent reaction. Under normal conditions, ALR2 has a central and fundamental role in the reduction of toxic aldehydes, originating from lipid peroxidation, and their adducts with glutathione, thus operating an important detoxifying and antioxidant action. In hyperglycaemic conditions, on the contrary, in cells where glucose up-take is independent of insulin, ALR2 operates the conversion of glucose into sorbitol leading to an accumulation of intracellular sorbitol. This determines an alteration of the osmolality and the oxidative stress with consequent production of reactive oxygen species (ROS) able of triggering ischaemic and inflammatory processes responsible for diabetic complications. The increase in sorbitol accumulation through the polyol pathway can lead to osmotic stress, which causes electrolyte imbalance, hydration and membrane damage. Decreased levels of NADPH can lead to oxidative stress and an increase in the NADH/NAD^+^ ratio can lead to reductive stress that causes pseudohypoxia with consequent cellular damage (Figure 1). Fructose phosphorylation can also lead to the formation of AGE (Advanced Glycation End-products) and the subsequent binding of AGEs to their receptors can lead to ROS production. Based on these considerations, reduction of the polyol pathway by the inhibition of the aldose reductase has long been considered a valid strategy to counteract or at least delay the onset of diabetic complications.

Diabetic retinopathy (DR) and cataract are considered the leading causes of blindness in diabetic patients. Many evidences indicate the involvement of polyol pathway in the pathophysiology of diabetic cataract^6^. Osmotic damage hypothesis postulate that the formation of cataracts is due to hyperosmotic effect caused by sorbitol accumulation in the cells as the result of excessive aldose reductase activity. The osmotic changes, moreover, causes cataracts formation by stimulation of apoptosis of lens epithelial cells^5^. ALR is also implicated with diabetic retinopathy that is a microvascular complication of diabetes mellitus characterised by retinal lesions, vascular damage and death or dysfunction of the neural retina^7^. In this context, the inhibition of the enzyme ALR2 is an emerging strategy for preventing and cure ocular diabetic complications. ALR2 is involved in diabetic retinopathy through numerous mechanisms but alterations in vascular permeability and oxidative stress can be prevented by the use of aldose reductase inhibitors (ARIs)^8^^,^^9^.

**Figure 2.**
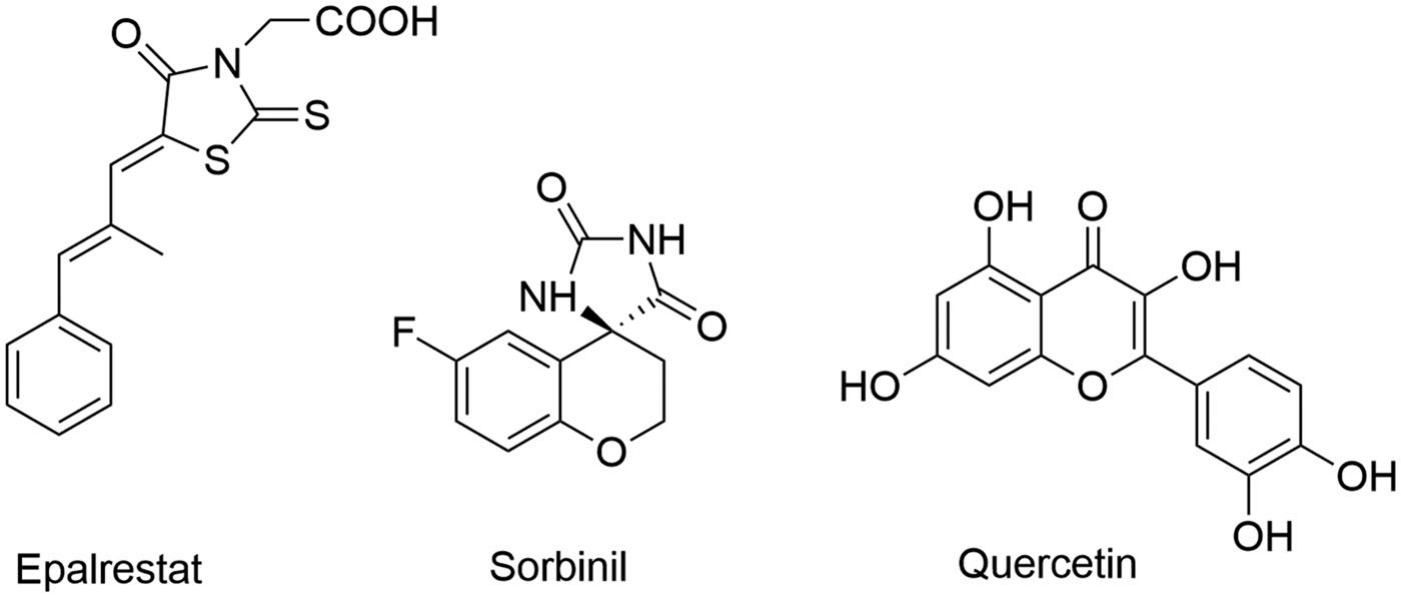
Structures of ARIs from different structural classes.

To date, a wide variety of structurally different compounds, both synthetic small molecules and natural compounds^10^^,^^11^, have shown inhibitory activity against aldose reductase (Aldose Reductase Inhibitors, ARIs) and many of these have been evaluated in preclinical and clinical trials^12^^,^^13^. Representative structural classes of ARIs include carboxylic acid derivatives, which are the largest and most important classes, cyclic imides, and phenolic derivatives (Figure 2)^5^^,^^14^.

Despite the chemical diversity of ARIs, they have two pharmacophoric portions: an acidic moiety which is able to fit well in the catalytic site of ALR2 interacting with the “anion-binding site” and a lipophilic scaffold which can bind to the highly flexible “specificity pocket” of the active site.

In search of new ARIs structurally different from the ones reported in literature, our previous work^15^ took in consideration some polyhydroxylated pyrrolidine derivatives as new chemotype of ALR2 inhibitors. The good results obtained suggested that the iminosugar scaffold represents a promising starting point for the design of new ALR2 inhibitors. Prompted by these results, in this work we designed a novel series of oxy-imino saccharidic derivatives by decorating the 1,5-dicarbonyl substrate with an arylidene-aminoxy moiety, to increase the chance of structural recognition with the enzyme binding site, characterised by a wide lipophilic portion.

The new derivatives were tested for the ability to inhibit ALR2 and the most promising compound was evaluated also for the ability to protect cells from hyperglycaemia-induced oxidative stress by evaluating targets such as Sod1, Sod2 and Nrf2. In fact, the ability to act as ALR2 inhibitors and, at the same time, as antioxidant represents in principle a synergistic strategy to slow down neurodegenerative mechanisms induced by chronic hyperglycaemia.

## 2. Experimental

### 2.1. Chemistry

Optical rotations were measured with an ATAGO AP-300 Automatic Polarimeter at 25 ± 2 °C. ^1^H NMR spectra were recorded in appropriate solvents with a Bruker Avance II operating at 250.13 MHz or 400 MHz (^1^H) and 62.9 MHz or 100 MHz (^13 ^C). The assignments were made, when possible, with the aid of DEPT-135, HSQC and COSY experiments. In the case of mixtures, assignments were made by referring to the differences in the peak intensities. The first order proton chemical shifts δ are referenced to either residual CD_3_CN (δ_H_ 1.94, δ_C_ 1.28) or residual CD_3_OD (δ_H_ 3.31 ppm, δ_C_ 49.0 ppm) and *J*-values are given in Hz. All reactions were followed by TLC on Kieselgel 60 F_254_ with detection by UV light and/or with ethanolic 10% phosphomolybdic or sulphuric acid, and heating or for exposure to iodine vapours. Kieselgel 60 (E. Merck, 230–400 mesh, respectively) was used for flash chromatography. Some of flash chromatography purifications were conducted by using Isolera Four SVTM (Biotage^®^), equipped with UV detector with variable wavelength (200–400 nm). Microwave-assisted reactions were run in a microwave synthesiser (Initiator^+^, Biotage^®^). All reactions involving air- or moisture-sensitive reagents were performed under an argon atmosphere using anhydrous solvents. All reagents, anhydrous MeOH, CH_2_Cl_2_ and DMF were purchased from Aldrich Chemical Co. and were used without further purification. MgSO_4_ was used as the drying agent for solutions. Elemental analysis was used to determine the purity of compounds. Analytical results are within ± 0.40% of the theoretical values.

“*Foam*” was referred to amorphous compounds, isolated pure by chromatography for which all attempts to crystallisation failed. Methyl 3,4-*O*-isopropylidene-6-*O*-tosyl-β-d-galactopyranoside was prepared according to the reported procedure^16^. *O*-(4-methoxybenzyl)hydroxylamine hydrochloride (**7a**)^17^ and *O*-(4-trifluoromethylbenzyl)hydroxylamine hydrochloride (**7 b**)^18^ were prepared according to literature by alkylation of *N*-hydroxyphtalimide with substituted benzyl bromide but the corresponding imides obtained were treated with NH_3_/MeOH 7 N instead of hydrazine hydrate and then with hydrochloride in ethyl ether to obtain the hydrochlorides **7a** and **7b**.

#### 2.1.1. Methyl 6-deoxy-3,4-O-isopropylidene-α-l-arabino-hex-5-enopyranoside (1)

A suspension of pre-washed (*n*-hexane) 60% NaH in mineral oil (618 mg, 25.7 mmol) in dry DMF (40 ml) was cooled to 0 °C and treated under argon atmosphere with a solution of the known methyl 3,4-*O*-isopropylidene-6-*O*-tosyl-β-d-galactopyranoside^16^ (2.00 g, 5.15 mmol) in dry DMF (40 ml). The mixture was gently warmed to room temperature, left under stirring until TLC analysis (1:1 *n*-hexane-EtOAc) revealed the complete disappearance of the starting material (*R*_f_ 0.18) and the formation of one spot (*R*_f_ 0.40) not visible under UV light. After 96 h, the reaction mixture was cooled to 0 °C, treated with crushed ice (50 ml) and extracted with Et_2_O (5 × 50 ml) and the combined organic phases were dried, filtered and concentrated at diminished pressure. Purification of the crude product (1.50 g) by flash chromatography on silica gel (1:1 *n*-hexane-EtOAc) using the Isolera Four Biotage^®^ system gave enol ether **1** (902 mg, 81% yield); as a solid foam; [α]_D_ -120.1 (c 1.2, CHCl_3_); *R*_f_ 0.21 (6:4 *n*-hexane-EtOAc); ^1^H NMR (400 MHz, CDCl_3_): δ 4.77 (bs, 1H, H-6b), 4.67 (bs, 1H, H-6a), 4.64 (d, 1H, *J*_3,4_ 6.9 Hz, H-4), 4.47 (d, 1H, *J*_1,2_ 7.1 Hz, H-1), 4.16 (t, 1H, *J*_2,3_ = *J*_3,4_ 6.9 Hz, H-3), 3.68 (ddd, 1H, *J*_2,3_ 6.8 Hz, *J*_2,OH_ 3.1 Hz, H-2), 3.53 (s, 3H, OMe), 2.93 (bs, 1H, OH), 1.50, 1.36 (2 s, each 3H, *Me_2_*C); ^13^C NMR (100 MHz, CDCl_3_): δ 153.3 (C-5), 111.5 (Me_2_*C*), 102.9 (C-1), 98.5 (C-6), 77.6 (C-3), 73.4, 72.6 (C-4, C-2), 57.3 (OMe), 28.0, 26.3 (Me_2_*C*). Anal. Calcd for C_10_H_16_O_5_: C, 55.55; H, 7.46. Found: C, 55.49; H, 7.41.

#### 2.1.2. 6-O-m-chlorobenzoyl-3,4-O-isopropylidene-l-arabino-hex-5-ulose (6a)

A solution of enol ether **1** (800 mg, 3.70 mmol, 1 eq) in dry CH_2_Cl_2_ (40 ml) was treated at 0 °C under argon atmosphere with a pre-dried solution (MgSO_4_) of 70% commercial MCPBA (1.09 g, 4.44 mmol, 1.2 eq) in dry CH_2_Cl_2_ (40 ml) and stirred at 0 °C until the starting material was completely disappeared (TLC, 7:3 *n*-hexane-EtOAc). After 2 h, TLC analysis showed the formation of one spot (*R*_f_ 0.35) visible under UV light and the reaction mixture was stirred for 1 h with anhydrous KF (1.03 g, 17.8 mmol, 4.8 eq) in order to eliminate the excess of MCPBA and MCBA. After filtration of the insoluble complex the organic solution was washed with saturated aq Na_2_CO_3_ (40 ml) and the aqueous phase was extracted with CH_2_Cl_2_ (4 × 30 ml). The combined organic extracts were dried, filtered, concentrated under diminished pressure and the residue (930 mg) was subjected to flash chromatography on silica gel (1:1 *n*-hexane-EtOAc) using the Isolera Four Biotage^®^ system. A foam constituted by a 9:1 mixture of the two C-5 anomeric aldehyde derivative **6a** (858 mg, 65% yield, NMR, CDCl_3_) was obtained; [α]_D_ +12.7 (c 1.1, CHCl_3_); *R*_f_ 0.21 (2:8 *n*-hexane-EtOAc); NMR data of the major C-5 anomer: ^1^H NMR (400 MHz, CDCl_3_): δ 9.63 (d, 1H, *J*_1,2_ 1.2 Hz, H-1), 8.04–7.96 (m, 2H, H-2′, H-6′ of *m*-Cl*Ph*CO), 7.55 (1 m, each 1H, H-4′ of *m*-Cl*Ph*CO), 7.40 (d, 1H, H-5′ of *m*-Cl*Ph*CO), 5.21 (dd, 1H, *J*_3,4_ 5.8 Hz, *J*_2,3_ 4.4 Hz, H-3), 4.68 (d, H, H-4), 4.59 (dd, 1H, H-2), 4.75, 4.60 (AB system, 2H, *J*_A,B_ 11.8 Hz, H-6a, H-6b), 1.44, 1.31 (2 s, each 3H, *Me*_2_C); ^13^C NMR (100 MHz, CDCl_3_) of major component: δ 197.6 (C-1), 165.7 (C=O), 134.6, 131.1 (2 × Ar-*C*), 133.4, 129.8, 129.7, 128.0 (4 × Ar-*C*H), 114.1 (Me_2_*C*), 104.9 (C-5), 84.3 (C-4), 83.7 (C-3), 81.5 (C-2), 65.3 (C-6), 25.8, 24.5 (*Me*_2_C). Selected NMR data of the minor C-5 anomer: ^1^H NMR (400 MHz, CDCl_3_): δ 9.61 (d, 1H, *J*_1,2_ 1.5 Hz, H-1), 5.16 (dd, 1H, *J*_3,4_ 6.2 Hz, *J*_2,3_ 4.8 Hz, H-3), 4.64 (m, H, H-4), 4.32 (dd, 1H, H-2), 4.50, 4.41 (AB system, 2H, *J*_A,B_ 11.7 Hz, H-6a, H-6b), 1.50, 1.37 (2 s, each 3H, *Me*_2_C); ^13^C NMR (100 MHz, CDCl_3_): δ 197.5 (C-1), 168.7 (C=O), 113.5 (Me_2_*C*), 104.1 (C-5), 84.7 (C-4), 80.5 (C-3), 79.7 (C-2), 65.3 (C-6), 25.8, 24.5 (*Me*_2_C). Anal. Calcd for C_16_H_17_ClO_7_: C, 53.87; H, 4.80. Found: C, 53.79; H, 4.71.

#### 2.1.3. Preparation of the mixture of O-(4-methoxybenzyl)-oxime derivatives (E/Z)-8

The aldehyde derivative **6a** (124.8 mg, 0.35 mmol, 1.0 eq) was treated with *O*-(4-methoxybenzyl)hydroxylamine hydrochloride **7a** (79.8 mg, 0.42 mmol, 1.2 eq) in a 3:1 CHCl_3_:H_2_O mixture (5.0 ml) and the solution was stirred at 60 °C. After 4 h, TLC analysis (6:4 *n*-hexane-EtOAc) revealed the complete disappearance of **6a** (*R*_f_ 0.11) and the formation of two spot (*R*_f_ 0.52 and 0.48). The solution was diluted with CHCl_3_ (5 ml), the aqueous phase extracted with CHCl_3_ (3 × 5 ml) and the combined organic phases were collected dried, filtered and concentrated at diminished pressure. Purification of the crude product (150 mg) by flash chromatography on silica gel (6:4 *n*-hexane-EtOAc) using the Isolera Four Biotage^®^ system gave a white foam (65.4 mg, 38% yield) constituted (NMR, CD_3_CN) by a mixture of isomers (*E*)-**8** and (*Z*)-**8** in a 63:37 ratio, measured on the relative intensities of the H-1 signals at δ 7.40 and 6.79 respectively.

The solution of **6a** (250 mg, 0.71 mmol, 1.0 eq) and *O*-(4-methoxybenzyl)hydroxylamine hydrochloride **7a** (159.5 mg, 0.84 mmol, 1.2 eq) in a 3:1 CHCl_3_:H_2_O mixture (10.0 ml) was stirred in a microwave sealed tube at 40 °C, and after 20 min, TLC analysis (6:4 *n*-hexane-EtOAc) revealed the complete disappearance of **6a** (*R*_f_ 0.11) and the formation of two spot (*R*_f_ 0.52 and 0.48). After the work-up described above, a solid foam was obtained (227 mg, 65% yield) which was constituted (NMR, CD_3_CN) by a mixture of isomers (*E*)-**8** and (*Z*)-**8** in a 65:35 ratio, measured on the relative intensities of the H-1 signals at δ 7.40 and 6.79 respectively. A second flash-chromatography on silica gel of a mixture (*E*)-**8** and (*Z*)-**8** (75:25 *n*-hexane-EtOAc) afforded pure samples pure of isomers (*E*)-**8** and (*Z*)-**8**.

Oxime (*E*)-**8**, solid foam, *R*_f_ 0.48 (6:4 *n*-hexane-EtOAc); ^1^H NMR (250.13 MHz, CD_3_CN): δ 8.06–7.93 (m, 2H, H-2′, H-6′ of *m*-Cl*Ph*CO), 7.66–7.62 (m, 1H, H-4′ of *m*-Cl*Ph*CO), 7.50 (m, 1H, H-5′ of *m*-Cl*Ph*CO), 7.40 (d, 1H, *J*_1,2_ 7.6 Hz, H-1), 7.30 (m, 2H, Ar-*H*,), 6.91 (m, 2H, Ar-*H*), 5.01 (s, 2H, C*H_2_*Ph), 4.92 (dd, 1H, *J*_3,4_ 5.8 Hz, *J*_2,3_ 4.0 Hz, H-3), 4.65 (d, 1H, H-4), 4.59 (dd, 1H, H-2), 4.46 (s, 2H, H-6a, H-6b), 4.36 (bs, 1H, OH-5), 3.78 (s, 3H, OMe), 1.43, 1.28 (2 s, each 3H, *Me*_2_C); ^13^C NMR (62.9 MHz, CD_3_CN): δ 165.6 (C=O), 160.5 (Ar-*C*-OMe), 147.6 (C-1), 135.0, 132.7, 130.5 (3 × Ar-*C*), 134.1–129.0 (Ar-*C*H), 114.9 (Ar-*C*H), 114.1 (Me_2_*C*), 104.5 (C-5), 86.3 (C-4), 82.9 (C-3), 77.7 (C-2), 76.5 (*C*H_2_PhOMe), 66.4 (C-6), 55.8 (OMe), 26.2, 24.8 (*Me*_2_C). Anal. Calcd for C_24_H_26_ClNO_8_: C, 58.60; H, 5.33; N, 2.85. Found: C, 58.56; H, 5.27; N, 2.79.

Oxime (*Z*)-**8**, solid foam, *R*_f_ 0.52 (6:4 *n*-hexane-EtOAc); ^1^H NMR (250.13 MHz, CD_3_CN): δ 8.07–7.92(m, 2H, H-2′, H-6′, of *m*-Cl*Ph*CO), 7.64 (m, 1H, H-4′ of *m*-Cl*Ph*CO), 7.50 (m, 1H, H-5′ of *m*-Cl*Ph*CO), 7.35–7.25 (m, 2H, Ar-*H*), 6.91 (m, 2H, Ar-*H*), 6.79 (bs, 1H, H-1), 5.11–4.99 (m, 4H, H-2, H-4, C*H_2_*Ph), 4.46 (m, 2H, H-6a, H-6b), 4.61 (m, 1H, H-3), 4.32 (bs, 1H, OH-5), 3.75 (s, 3H, OMe), 1.43, 1.27 (2 s, each 3H, *Me*_2_C); ^13^C NMR (62.9 MHz, CD_3_CN): δ 165.6 (C=O), 160.5 (Ar-*C*-OMe), 149.0 (C-1), 135.0, 132.8, 131.3 (3 × Ar-C), 134.1–128.9 (Ar-*C*H), 114.6 (Ar-*C*H), 113.7 (Me_2_*C*), 104.6 (C-5), 85.7 (C-4), 81.8 (C-3), 75.6 (C-2), 76.7 (*C*H_2_PhOMe), 66.4 (C-6), 55.8 (OMe), 26.3, 24.9 (*Me*_2_C). Anal. Calcd for C_24_H_26_ClNO_8_: C, 58.60; H, 5.33; N, 2.85. Found: C, 58.57; H, 5.29; N, 2.80.

#### 2.1.4. Preparation of the mixture of O-(4-trifluoromethylbenzyl)-oxime derivatives (E/Z)-9

*A* solution of aldehyde derivative **6a** (93.8 mg, 0.263 mmol, 1.0 eq) and *O*-(4-trifluoromethylbenzyl)hydroxylamine hydrochloride **7 b** (59.8 mg, 0.263 mmol, 1.0 eq) was treated under MW irradiation as described above for the preparation of (*E/Z*)-**8**. The reaction mixture was stirred in a microwave sealed tube at 40 °C until TLC analysis (1:1 *n*-hexane-EtOAc) revealed the complete disappearance of the starting material **6a** (*R*_f_ 0.15) and the formation of a single spot (*R*_f_ 0.64). After work-up, the crude product (130 mg) was purified by flash chromatography on silica gel (8:2 n-hexane-EtOAc) using the Isolera Four Biotage^®^ system to give a clear syrup (83 mg, 60% yield) constituted (NMR, CD_3_CN) by a mixture of isomers (*E*)-**9** and (*Z*)-**9** in a 60:40 ratio, measured on the relative intensities of the H-1 signals at δ 7.48 and 6.84 respectively. A second purification of a mixture of (*E*)-**9** and (*Z*)-**9** by flash-chromatography on silica gel (85:25 *n*-hexane-EtOAc) afforded a sample constituted (^1^H NMR) by a mixture of isomers (*E*)-**9** and (*Z*)-**9** in the ratio of 93:7. Compounds (*E*)-**9** and (*Z*)-**9** were inseparable by TLC with several elution systems and their structures were confirmed from their NMR data. ^1^H NMR (250.13 MHz, CD_3_CN) of oxime (*E*)-**9**: δ 7.48 (d, 1H, *J*_1,2_ 7.8 Hz, H-1), 5.20 (s, 2H, C*H_2_*PhCF_3_), 4.92 (dd, 1H, *J*_3,4_ 5.8 Hz, *J*_2,3_ 4.0 Hz, H-3), 4.65 (d, 1H, H-4), 4.59 (dd, 1H, H-2,), 4.36 (bs, 1H, OH-5), 4.46 (s, 2H, H-6a, H-6b), 1.43, 1.29 (2 s, each 3H, *Me*_2_C); of oxime (*Z*)-**9**: δ 6.84 (d, 1H, *J*_1,2_ 3.7 Hz, H-1), 5.23 (s, 2H, CH_2_PhCF_3_), 5.18–5.10 (m, 2H, H-2, H-3), 4.62 (d, 1H, *J*_3,4_ 4.6 Hz, H-3), 4.46 (s, 2H, H-6a, H-6b), 1.40, 1.29 (2 s, each 3H, *Me*_2_C); cluster of signals for (*E*)-**9** and (*Z*)-**9**: δ 8.10–7.85 and 7.73–7.49 (2 m, 8H, *m*-Cl*Ph*CO, Ar-*H*); ^13^C NMR (62.9 MHz, CD_3_CN) of oxime (*E*)-**9**: δ 148.6 (C-1), 114.1 (Me_2_*C*), 105.0 (C-5), 86.3 (C-4), 82.8 (C-3), 77.6 (C-2), 75.7 (*C*H_2_PhCF_3_), 26.2, 24.8 (*Me*_2_C); of oxime (*Z*)-**9**: δ 149.9 (C-1), 113.8 (Me_2_*C*), 104.6 (C-5), 85.7 (C-4), 81.8 (C-3), 76.9 (*C*H_2_PhCF_3_), 75.8 (C-2), 26.3, 24.9 (*Me*_2_C); cluster of signals for (E)-**9** and (Z)-**9**: δ 165.6 (C=O), 143.5, 135.0, 131.3 (3 × Ar-*C*), 134.1–128.9 (Ar-*C*H), 126.2 (Ar-*C*H), 66.3 (C-6). Anal. Calcd for C_24_H_23_ClF_3_NO_7_: C, 54.40; H, 4.38; N, 2.64. Found: C, 54.34; H, 4.35; N, 2.60.

#### 2.1.5. General procedure for the reduction of the mixture of oxime derivatives (E/Z)-8–11

##### Method A (with NaBH_4_, dry MeOH)

A solution of opportune oxime derivatives (*E/Z*)-**8** or (*E/Z*)-**9** (1.0 eq) in dry MeOH (10.0 ml) was cooled to 0 °C and treated with NaBH_4_ (3.0 eq) and the solution was stirred at room temperature (1 h) and then at 40 °C (1 h) until TLC analysis (6:4 *n*-hexane-EtOAc) showed complete disappearance of the starting material. MeOH was evaporated under reduced pressure, and the residue was partitioned with CH_2_Cl_2_ (15 ml) and H_2_O (15 ml), the phases were separated and the aqueous one was further extracted with CH_2_Cl_2_ (3 × 15 ml). The combined organic phases were dried, filtered and concentrated under diminished pressure. The crude product was purified by flash chromatography on silica gel using the Isolera Four Biotage^®^ system.

##### Method B (with LiAlH_4_, dry Et_2_O)

A solution of the selected mixture of (*E/Z*)-**10** or (*E/Z*)-**11** (1.0 eq) in dry Et_2_O (10 ml) was slowly added under Argon, to a suspension of LiAlH_4_ (10 eq) in Et_2_O (5.0 ml) cooled to 0 °C and the mixture was stirred at room temperature for 2 h. Unreacted hydride was decomposed by addition of H_2_O (0.40 ml), then aqueous 15% NaOH (1.20 ml), and H_2_O (0.40 ml). The mixture was stirred for 15 min, filtered, repeatedly washed with Et_2_O and the combined organic phases were dried, filtered and concentrated under diminished pressure. The crude product was purified by flash chromatography on silica gel using the Isolera Four Biotage^®^ system.

##### Method C (with NaBH_3_CN, AcOH, dry MeOH, 60 °C)

To a solution of the opportune oxime derivatives (*E/Z*)-**8** or (*E/Z*)-**9** (1.0 eq) in dry MeOH (40 ml), glacial AcOH (0.28 ml) and then a solution of NaBH_3_CN (4.0 eq) in dry MeOH (25 ml) were added. The mixture was heated to 60 °C and stirred until the starting material was completely disappeared (TLC analysis, 3–4 days). The reaction mixture was cooled to room temperature, neutralised by addition of Et_3_N and concentrated under diminished pressure. The crude product was purified by flash chromatography on silica gel using the Isolera Four Biotage^®^ system.

##### Method D (with NaBH_3_CN, AcOH, dry MeOH, 60 °C, MW irradiation)

To a solution of the opportune oxime derivatives (*E/Z*)-**8** or (*E/Z*)-**9** (1.0 eq) in dry MeOH (20 ml), glacial AcOH (0.26 ml) and NaBH_3_CN (3.0 eq) were added. The solution was stirred in a microwave sealed tube at 60 °C until the starting material was completely disappeared (TLC analysis, 1.5–2 h). The reaction mixture was cooled to room temperature, neutralised by addition of Et_3_N and concentrated under diminished pressure. The crude product was purified by flash chromatography on silica gel using the Isolera Four Biotage^®^ system.

#### 2.1.6. Preparation of the mixture of 6-O-deprotected oxime derivatives (E/Z)-10

A solution of (*E/Z*)-**8** (*E*:*Z* = 65:35) (51.2 mg, 0.104 mmol, 1 eq) in dry MeOH (2.0 ml) was treated with NaBH_4_ (11.9 mg, 0.312 mmol, 3 eq) according to the general procedure (Method A). Purification of the crude product (41 mg) by flash chromatography on silica gel (6:4 *n*-hexane-EtOAc) afforded a pure a syrup (26 mg, 70% yield) constituted (NMR,) by a mixture of isomers (*E*)-**10** and (*Z*)-**10** in a 55:45 ratio, measured on the relative intensities of the H-1 signals at δ 7.36 and 6.74 respectively. Compounds (*E*)-**10** and (*Z*)-**10** were inseparable by TLC (*R*_f_ 0.14, 6:4 *n*-hexane-EtOAc) with several elution systems and their structures were confirmed from their NMR data. ^1^H NMR (400 MHz, CD_3_CN) of oxime (*E*)-**10**: δ 7.36 (d, 1H, *J*_1,2_ 7.6 Hz, H-1), 5.00 (s, 2H, C*H_2_*Ph), 4.84 (dd, 1H, *J*_3,4_ 5.8 Hz, *J*_2,3_ 4.1 Hz, H-3), 4.52 (d, 1H, H-4), 4.49 (dd, 1H, H-2), 3.78 (s, 3H, OMe), 3.69–3.55 (m, 4H, H-6b, H-6a, 2 × OH), 1.40, 1.26 (2 s, each 3H, *Me*_2_C); of oxime (*Z*)-**10**: δ 6.74 (d, 1H, *J*_1,2_ 4.0 Hz, H-1), 5.04 (s, 2H, C*H_2_*Ph), 4.99–4.95 (m, 2H, H-2, H-4), 4.47 (d, 1H, *J*_3,4_ 5.4 Hz, H-3), 4.04–3.94 (m, 2H, H-6a, H-6b), 3.78 (s, 3H, OMe), 3.00–2.90 (bs, 1H, 2 × OH), 1.37, 1.25 (2 s, each 3H, *Me*_2_C); cluster of signals for (*E*)-**10** and (*Z*)-**10**: δ 7.38–7.28 (m, 2H, Ar-*H*), 6.91 (m, 2H, Ar-*H*); ^13^C NMR (100 MHz, CD_3_CN) of oxime (*E*)-**10**: δ 149.5 (C-1), 113.7 (Me_2_*C*), 106.1 (C-5), 86.0 (C-4), 82.9 (C-3), 77.2 (C-2), 76.6 (*C*H_2_PhOMe), 26.3, 24.9 (*Me*_2_C); of oxime (*Z*)-**11**: δ 148.0 (C-1), 113.4 (Me_2_*C*), 105.7 (C-5), 85.5 (C-4), 81.8 (C-3), 75.1 (C-2), 76.4 (*C*H_2_PhOMe), 26.1, 24.7 (*Me*_2_C); cluster of signals for (*E*)-**10** and (*Z*)-**10**: δ 160.3 (Ar-*C*-OMe), 139.3 (Ar-*C*), 131.0–130.7 (Ar-*C*H), 114.7 (Ar-*C*H), 63.9 (C-6), 55.8 (OMe). Anal. Calcd for C_17_H_23_NO_7_: C, 57.78; H, 6.56; N, 3.96. Found: C, 57.74; H, 6.51; N, 3.92.

#### 2.1.7. Preparation of the mixture of the 6-O-deprotected oxime derivatives (E/Z)-11

The reduction of mixture *(E/Z)-****10*** (E:Z = 60:40) (58.4 mg, 0.11 mmol, 1 eq) was performed in dry MeOH (2.2 ml) with NaBH_4_ (12.5 mg, 0.496 mmol, 3 eq), as described in the general procedure (Method A). Purification of crude product (45 mg) by flash chromatography on silica gel (6:4 *n*-hexane-EtOAc) afforded a pure syrup (27 mg, 63% yield) constituted (NMR) by a mixture of isomers (*E*)-**11** and (*Z*)-**11** in the ratio of 60:40, measured on the relative intensities of the H-1 signals at δ 7.46 and 6.80 respectively. Compounds (*E*)-**11** and (*Z*)-**11** were inseparable on TLC (*R*_f_ 0.13, 6:4 *n*-hexane-EtOAc) with several elution systems and their structures were confirmed from their NMR data. ^1^H NMR (400 MHz, CD_3_OD) of oxime (*E*)-**11**: δ 7.46 (d, 1H, *J*_1,2_ 7.5 Hz, H-1), 5.17 (s, 2H, C*H_2_*Ph), 4.86 (dd, 1H, *J*_3,4_ 5.8 Hz, *J*_2,3_ 4.0 Hz, H-3), 4.57 (dd, 1H, H-2), 4.55 (d, 1H, H-4), 1.47, 1.29 (2 s, each 3H, *Me*_2_C); of oxime (*Z*)-**11**: δ 6.80 (d, 1H, *J*_1,2_ 4.0 Hz, H-1), 5.22 (s, 2H, C*H_2_*Ph), 5.11–5.04 (m, 2H, H-2, H-4), 4.53 (d, 1H, *J*_3,4_ 5.2 Hz, H-3), 1.40, 1.28 (2 s, each 3H, *Me*_2_C); cluster of signals for (*E*)-**11** and (*Z*)-**11**: δ 7.65–7.62 (m, 2H, Ar-*H*), 7.56–7.50 (m, 3H, Ar-*H*, OH), 3.68–3.57 (m, 2H, H-6a, H-6b); ^13^C NMR (100 MHz, CD_3_CN) of oxime (*E*)-**11**: δ 149.0 (C-1), 143.5 (Ar-*C*), 113.7 (Me_2_*C*), 106.2 (C-5), 86.0 (C-4), 82.8 (C-3), 77.0 (C-2), 75.6 (*C*H_2_PhO), 63.8 (C-6), 26.1, 24.7 (*Me*_2_C); of oxime (*Z*)-**11**: δ 150.4 (C-1), 143.8 (Ar-*C*), 113.3 (Me_2_*C*), 105.8 (C-5), 85.5 (C-4), 81.8 (C-3), 75.8 (*C*H_2_PhOMe), 75.4 (C-2), 63.8 (C-6), 26.3, 24.9 (*Me*_2_C); cluster of signals for (*E*)-**11** and (*Z*)-**11**: δ 129.4–129.0 (Ar-*C*H, Ar-*C*), 126.2 (Ar-*C*H). Anal. Calcd for C_17_H_20_F_3_NO_6_: C, 52.18; H, 5.15; N, 3.58. Found: C, 52.15; H, 5.12; N, 3.54.

#### 2.1.8. Reduction of the mixture of (E/Z)-10 or (E/Z)-11 with LiAlH_4_

The reduction of the either mixture (*E/Z)-***10** (*E*:*Z* = 55:45, 0.1 mmol) or (*E/Z*)-**11** (*E*:*Z* = 60:40, 0.1 mmol) was performed in dry Et_2_O (2.5 ml) and LiAIH_4_ (1.0 mmol, 10 eq), as described in the general procedure (Method B). The crude residue was constituted by the starting material (*E/Z*)-**10** or (*E/Z*)-**11** as showed by NMR analysis.

#### 2.1.9. Reduction of the mixture of (E/Z)-8 with NaBH_3_CN

The reduction of mixture *(E/Z)-****8*** (*E*:*Z* = 60:40) (198 mg, 0.40 mmol, 1 eq) was performed in dry MeOH (28 ml) with glacial AcOH (153 µL) and NaBH_3_CN (100.6 mg, 1.60 mmol, 4 eq), as described in the general procedure (Method C). After 6 days, the TLC analysis (1:1 n-hexane-EtOAc) revealed of the starting material (*E/Z*)-**8** (*R*_f_ 0.15) and the formation of a major single spot (*R*_f_ 0.38). After work-up and purification of the crude product by flash chromatography on silica gel (6:4 *n*-hexane-EtOAc) a starting material (*E/Z*)-**8** (49.4 mg, 25% yield) and the 6-*O*-(*m*-chlorobenzoyl)-3,4-*O*-isopropylidene-*N*-(*p*-methoxybenzyloxy)-1,5-dideoxy-1,5-imino-d-galactitol (**12**) (86.0 mg, 45% yield) were isolated. Compound **12**, clear syrup; *R*_f_ 0.20 (1:1 *n*-hexane-EtOAc); ^1^H NMR (400 MHz, CD_3_CN): δ 7.95 (bt, 1H, *J* 1.6 Hz, H-2′ of *m*-Cl*Ph*CO), 7.90 (dt, 1H, *J* 1.1 Hz, *J* 7.7 Hz, H-6′ of *m*-Cl*Ph*CO), 7.62 (m, 1H, H-4′ of *m*-Cl*Ph*CO), 7.46 (dd, 1H, *J* 7.7 Hz, *J* 8.0 Hz, H-5′ of *m*-Cl*Ph*CO), 7.27 (m, 2H, Ar-*H*,), 6.87 (m, 2H, Ar-*H*), 4.74 (dd, 1H, *J*_6a,6b_ 10.8 Hz, *J*_5,6b_ 5.3 Hz, H-6b), 4.66, 4.57 (AB system, 2 H, *J*_A,B_ 10.2 Hz, CH_2_Ph), 4.46 (dd, 1H, *J*_5,6a_ 7.4 Hz, H-6a), 4.41 (dd, 1H, *J*_4,5_ 3.8 Hz, *J*_3,4_ 5.0 Hz, H-4), 3.83–3.71 (m, 2H, H-2, H-3), 3.75 (s, 3H, OMe), 3.38 (dd, 1H, J_1ax,1eq_ 11.8 Hz, J_1eq,2_ 4.0 Hz, H-1eq), 3.31 (bs, 1H, OH-2), 3.20 (ddd, 1H, H-5), 2.36 (dd, 1H, J_1ax,2_ 10.5 Hz, H-1ax), 1.43 e 1.27 (2 s, each 3H, *Me*_2_C); ^13^C NMR (100 MHz, CD_3_CN): δ 165.7 (C=O), 160.5 (Ar-*C*-OMe), 135.6, 133.3, 131.2 (3 × Ar-*C*), 134.2–128.7 (Ar-*C*H), 114.5 (Ar-*C*H), 109.7 (Me_2_*C*), 80.2, 69.9 (C-2, C-3), 75.4 (*C*H_2_PhOMe), 75.1 (C-4), 64.4 (C-6); 64.2 (C-5), 59.3 (C-1), 55.8 (OMe), 28.5, 26.2 (*Me*_2_C). Anal. Calcd for C_24_H_28_ClNO_7_: C, 60.31; H, 5.91; N, 2.93. Found: C, 60.27; H, 5.87; N, 2.88.

An identical result was obtained when the reduction of mixture (E/Z)-***8*** (98 mg, 0.20 mmol, 1 eq) was conducted in a microwave sealed tube at 60 °C (2 h), as described in the general procedure (Method D). Purification of the crude product by flash chromatography on silica gel (6:4 *n*-hexane-EtOAc) gave (*E/Z*)-**8** (34.4 mg, 35% yield) and pure **12** (31.5 mg, 33% yield).

#### 2.1.10. Reduction of the mixture of (E/Z)-9 with NaBH_3_CN

The reduction of mixture *(E/Z)-****9*** (*E*:*Z* = 60:40) (49.6 mg, 0.094 mmol, 1.0 eq) was performed in a microwave sealed tube at 60 °C (1.5 h) with MeOH (3.0 ml), glacial AcOH (24 µL) and NaBH_3_CN (17.6 mg, 0.28 mmol, 3.0 eq), as described in the general procedure (Method D). After work-up of reaction, NMR analysis showed that the crude product constituted only by starting material (*E/Z*)-**9**.

#### 2.1.11. Double reductive amination of l-arabino-hexos-5-ulose derivative (6a) with 7a

*Method A* (*NaBH_3_CN, dry MeOH, 60 °C)*. A solution of aldehyde derivative **6a** (200 mg, 0.56 mmol, 1.0 eq) in dry MeOH (11 ml) was treated with *O*-(4-methoxybenzyl)hydroxylamine hydrochloride **7a** (117 mg, 0.62 mmol, 1.1 eq) and NaBH_3_CN (77.5 mg, 1.23 mmol, 2.2 eq). The mixture was stirred at 60 °C (96 h) until TLC analysis (4:6 *n*-hexane-EtOAc) revealed the complete disappearance of the starting material **6a** (*R*_f_ 0.59) and the formation of three spots (*R*_f_ 0.50, 0.42 and 0.24). The solution was concentrated under diminished pressure, the residue was treated with CH_2_Cl_2_ (20 ml) and satd aq NaHCO_3_ solution (20 ml), the aqueous phase was extracted with CH_2_Cl_2_ (4 × 30 ml), and the organic layers were collected, dried and concentrated under diminished pressure. Purification of crude product by flash chromatography on silica gel (6:4 hexane-EtOAc) using the Isolera Four Biotage^®^ system gave pure **12** (53.5 mg, 20%), a mixture of pure (*E/Z*)-**8** (27.5 mg, 10%) and a mixture of pure (*E/Z)-***10** (79 mg, 40%).

*Method B* (*NaBH_3_CN, dry MeOH, AcOH pH 5–6, 60 °C)*. Double reductive amination of **6a** (100 mg, 0.28 mmol, 1.0 eq) with **7a** (58.5 mg, 0.31 mmol, 1.1 eq) was performed in dry MeOH (5.5 ml), NaBH_3_CN (38.8 mg, 0.62 mmol, 2.2 eq) in the presence of glacial AcOH (110 µL) for 96 h, according to the procedure described above. Purification of the crude product by flash chromatography on silica gel eluted with 6:4 hexane-EtOAc) gave pure **12** (22 mg, 15%), a mixture of (*E/Z*)-**8** (18.3 mg, 12%) and a mixture of (*E/Z*)-**10** (47 mg, 43%).

*Method C* (*NaBH_3_CN, dry MeOH, AcOH pH 5–6, 40 °C, MW irradiation)*. To a solution of **6a** (100 mg, 0.28 mmol, 1.0 eq) in dry MeOH (3.0 ml), **7a** (58.3 mg, 0.31 mmol, 1.1 eq), NaBH_3_CN (52.8 mg, 0.54 mmol, 3.0 eq) and glacial AcOH (73 µL) were added. The solution was stirred in a microwave sealed tube at 40 °C until the starting material was completely disappeared (TLC analysis, 30 min). Treatment of the reaction and purification of the crude product by flash chromatography on silica gel (6:4 hexane-EtOAc), according to the procedure described above, gave pure **12** (33 mg, 25%), a mixture of (*E/Z*)-**8** (18.3 mg, 22%) and a mixture of (*E/Z*)-**10** (10.9 mg, 11%).

#### 2.1.12. Double reductive amination of l-arabino-hexos-5-ulose derivative (6a) with 7 b

To a solution of **6a** (45.7 mg, 0.128 mmol, 1.0 eq) in dry MeOH (2.0 ml), **7b** (35 mg, 0.154 mmol, 1.2 eq), NaBH_3_CN (29.1 mg, 0.46 mmol, 3.8 eq) and glacial AcOH (73 µL) were added. The solution was stirred in a microwave sealed tube at 40 °C until the starting material was completely disappeared (TLC, 1:1 hexane-EtOAc, 2 h). Treatment of the reaction and purification of the crude product by flash chromatography on silica gel (1:1 hexane-EtOAc), in accordance to the procedure described above, gave a pure mixture of (*E/Z*)-**9** (37.3 mg, 55%).

### 2.2. Biological assays

Human recombinant aldose reductase from *Escherichia coli*, d,l-glyceraldehyde, NADPH, NaH_2_PO_4_, Na_2_HPO_4_ and Tolrestat were purchased from Sigma Aldrich.

#### 2.2.1. ALR2 enzymatic inhibition in cell-free assays

ALR2 activity was determined spectrophotometrically at 340 nm by monitoring the change in absorbance of the NADPH cofactor in presence of the test compounds, **8–10**, following a previously reported procedure.^15^ Compounds were initially solubilised in DMSO, then diluted with water to test concentration. DMSO concentration in the test solutions never exceed 4% and proved to have no inhibitory effects on the target enzyme. Assays were conducted in triplicate and Tolrestat was used as the reference compound.

#### 2.2.2. ALR2 enzymatic inhibition in cell-based assays

ALR2 activity was determined in a 661 W cell line, derived from immortalised cone photoreceptors (provided by Muayyad Al-Ubaidi, University of Oklahoma), which was cultured in 24.5 mM (NG) or 55 mM (HG) glucose-containing medium, in the absence or presence of (*Z*)-**8** (50 and 100 µM) for 24 h, following a previously described procedure^15^ (see Supplementary Material for full experimental details).

#### 2.2.3. Cellular viability, detection of apoptosis, immunocytochemistry studies and Western blot analysis in 661Wcell line

The 661 W cell line was cultured in 24.5 mM (NG) or 55 mM (HG) glucose-containing medium, in the absence or presence of (*Z*)-**8** (50 and 100 µM) for 24 h, as previously reported.^15^ Quantification of cellular viability and apoptosis, immunochemistry tests and Wester blot analysis were performed as already described^15^ (see Supplementary Material for full experimental details).

## 3. Results and discussion

### 3.1. Chemistry

**Figure 3.**
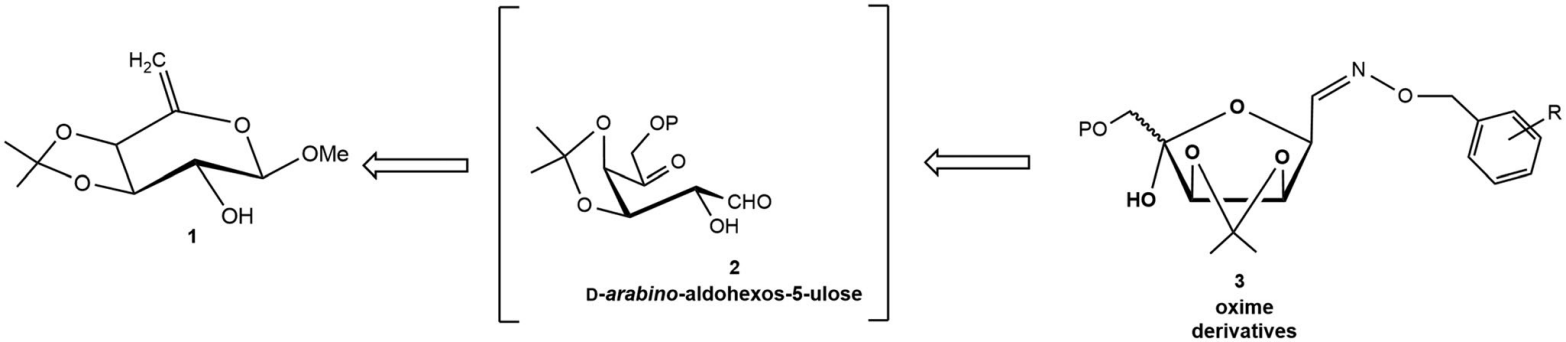
Retrosynthetic approach to the oxime derivatives **3**.

Aldohexos-5-uloses (type **2**) are versatile platform chemicals for accessing biologically relevant targets such as iminosugars^15^^,^^16^^,^^19^ and cyclitols^20^. As a continuation of our recent results aimed at searching new potential ALR2 inhibitors^15^ from these 1,5-dicarbonyl substrates, we studied their reaction with *O*-(arylmethyl)hydroxylamines hydrochlorides (Figure 3).

**Scheme 1.**
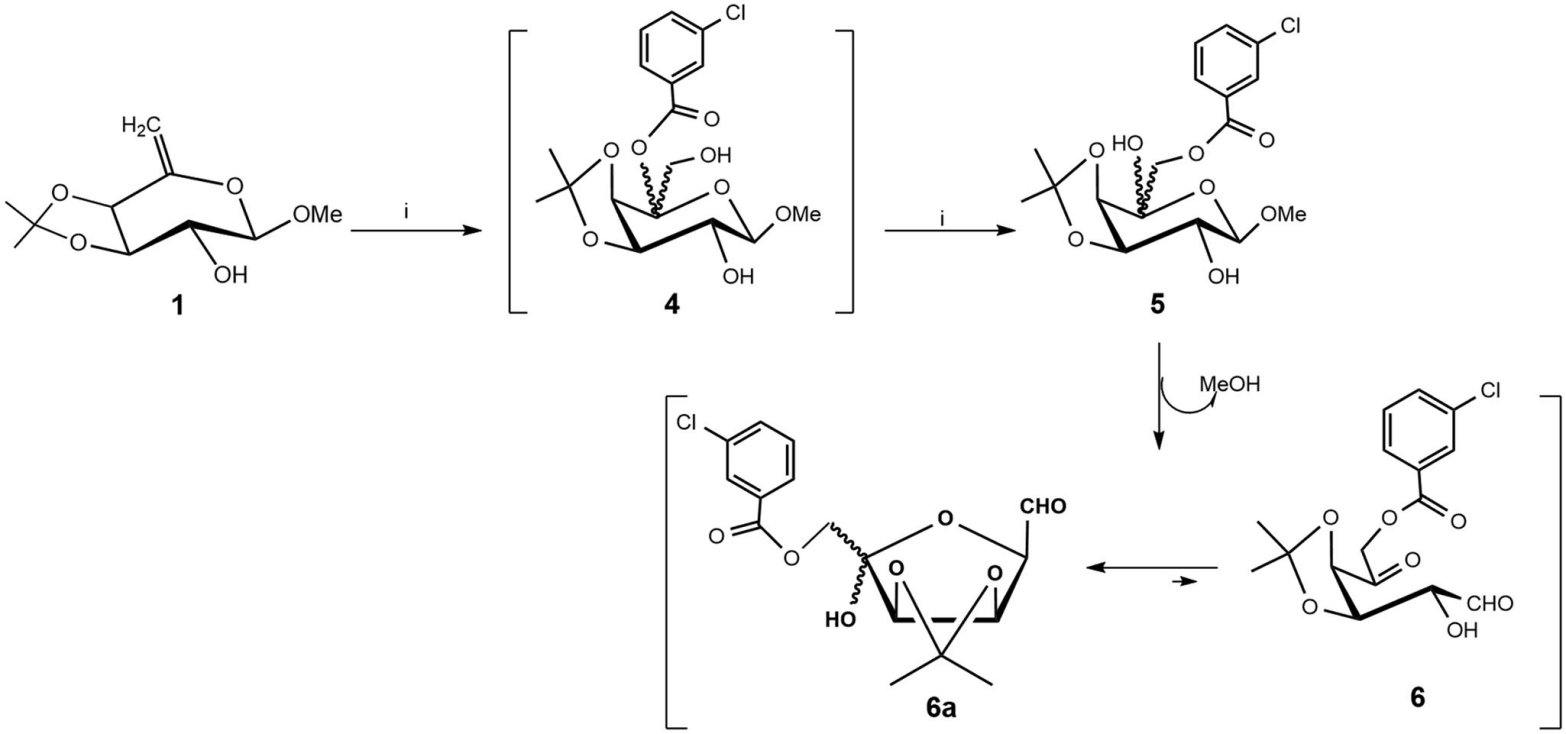
Reagents and conditions: (i) MCPBA, dry CH_2_Cl_2_, rt, 2 h and then anhydrous KF (**6a**: 65% yield from **1**).

The 6-deoxy-hex-5-enol ether **1** (Scheme 1) was subjected to an epoxidation reaction with meta-chloroperoxybenzoic acid (MCPBA) in dry CH_2_Cl_2_ leading, after treatment with dry KF, usual work-up and chromatographic purification on silica gel, to the d-*arabino*-aldohexos-5-ulose derivative **6** (63% isolated yield). This result can be explained by the regioselective attack of the nucleophile (MCBO^−^) at C-5 on the transient protonated epoxide to give the 5-*m*-chlorobenzoate derivative **4**, followed by an intramolecular acyl migration, promoted by fluoride ions^21^, from the tertiary *O*-5 to the primary *O*-6 with formation the 6-*m*-chlorobenzoate intermediate **5**. This spontaneous acyl migration was observed during our previous studies on the epoxidation of hex-5-enopyranosides^22^ and hex-3-enofuranosides^23^. Finally, the formation of 1,5-dicarbonyl derivative **6** was obtained from the 6-*m*-chlorobenzoate **5** by spontaneous elimination of MeOH. The NMR analysis (CDCl_3_, see Experimental Section and Supplementary Material) showed that the tautomeric equilibrium of **6** is characterised by a 9:1 mixture of the two C-5 anomeric aldehyde derivatives **6a** (Scheme 1) deriving from the intramolecular hemiacetalisation of the 2-OH on the 5-*keto* group. In particular, in the proton spectrum (Table 1), the only presence of a doublet at δ 9.63 (*J*_1,2_ =1.04 Hz), and two double doublets at δ 5.21 (*J*_3,4_ =5.8 Hz) and 4.59 (*J*_2,3_ =4.4 Hz), related to aldehyde, H-3 and H-2 protons, confirmed the exclusive presence of furanose tautomer **6a**. The low values of these coupling constants strongly suggest the absence of pyranose tautomers. The d-*arabino*-aldohexos-5-ulose derivative **6a** can be stored at -20 °C, under argon atmosphere for several months.

**Table 1.** Selected ^1^H NMR data (δ, ppm) of d-*arabino*-aldohexos-5-ulose derivative **6a** and of arylmethyloxyimino derivatives **8–11**.

Compound	Solvent	H-1	H-2	H-3	H-4	H-6a, H-6b	OH
**6a**	CDCl_3_	9.63	4.59	5.21	4.68	4.75, 4.60	–
**(*E*)-8**	CD_3_CN	7.40	4.59	4.92	4.65	4.46	4.36
**(*Z*)-8**	CD_3_CN	6.79	5.11–4.99	4.61	5.11–4.99	4.46	4.32
**(*E*)-9**^a^	CD_3_CN	7.48	4.59	4.92	4.65	4.46	4.36
**(*Z*)-9**^a^	CD_3_CN	6.84	5.18–5.10	4.62	5.18–5.10	4.46	–
**(*E*)-10**^a^	CD_3_CN	7.36	4.49	4.84	4.52	3.65–3.58	3.65–3.55
**(*Z*)-10**^a^	CD_3_CN	6.74	4.99–4.95	4.47	4.99–4.95	4.03–3.94	4.31
**(*E*)-11**^a^	CD_3_OD	7.46	4.57	4.86	4.55	3.68–3.57	–
(*Z*)-11^a^	CD_3_OD	6.80	5.11–5.04	5.53	5.11–5.04	3.68–3.57	–

^a^ NMR data were obtained from analysis of isomeric mixture.

**Scheme 2.**
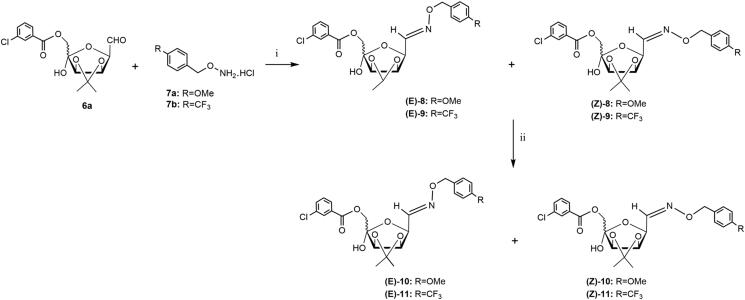
Reagents and conditions: (i) 3:1 CHCl_3_-H_2_O, 60 °C, 4 h (*E/Z*-**8**: 38%); or 3:1 CHCl_3_-H_2_O, MW irradiation, 40 °C, 20 min (*E/Z*-**8**: 65%; *E/Z*-**9**: 60%); (ii) NaBH_4_, dry MeOH, rt, 1 h, and then at 40 °C, 1 h (*E/Z*-**10**: 70%; *E/Z*-**11**: 63%).

The reaction of derivative **6a** (Scheme 2) with the *O*-(4-methoxybenzyl)hydroxylamine hydrochloride **7a**^17^ in a 3:1 CHCl_3_-H_2_O mixture at 60 °C (4 h) afforded, after purification by flash chromatography the corresponding arylmethyloxyimino derivatives **8** in low yield as mixture of *E/Z* isomers [38% (*E*)-**8**:(*Z*)-**8 **=** **63:37]. To reduce reaction times and increase yields, the reactions of **6a** (Scheme 2) with **7a**^17^ or **7b**^18^ was performed in a 3:1 CHCl_3_-H_2_O mixture under MW irradiation at 40 °C (20 min). The purification by flash chromatography on silica gel of crude products gave the corresponding oxime derivatives **8** and **9** in satisfactory yields as mixtures of *E/Z* isomers [65% (*E*)-**8**:(*Z*)-**8 **=** **65:35; 60% (*E*)-**9**:(*Z*)-**9 **=** **60:40]. Only in the case of the (*E/Z*)-**8** mixture, a partial separation of the two isomers (*E*)-**8** and (*Z*)-**8** by flash chromatography on silica gel was possible.

Although the reaction between *N*-alkyl-oxyamines and unprotected carbohydrates (aldohexoses) to give the acyclic oximes (*E*- and *Z*-configuration) in equilibrium with the corresponding cyclic *N*-glycosides has already been reported in the literature^24^^,^^25^, no data have been found for aldohexos-5-uloses derivatives.

Attempts to prepare the corresponding aryloxyamino derivatives by reduction of the isomeric mixtures of (*E/Z*)-**8** or (*E/Z*)-**9** with NaBH_4_ in dry MeOH (Scheme 2) failed, and in all cases the corresponding 6-*O*-deprotected derivatives **10** and **11** were isolated. Purification by flash chromatography on silica gel of crude products afforded pure **10** and **11** as mixtures of *E/Z* isomers (NMR) in a good yield (70 and 63% respectively). Moreover, the isomeric mixtures (*E/Z*)-**10** or (*E/Z*)-**11** were recovered unchanged after treatment with LiAlH_4_ in dry Et_2_O.

**Scheme 3.**
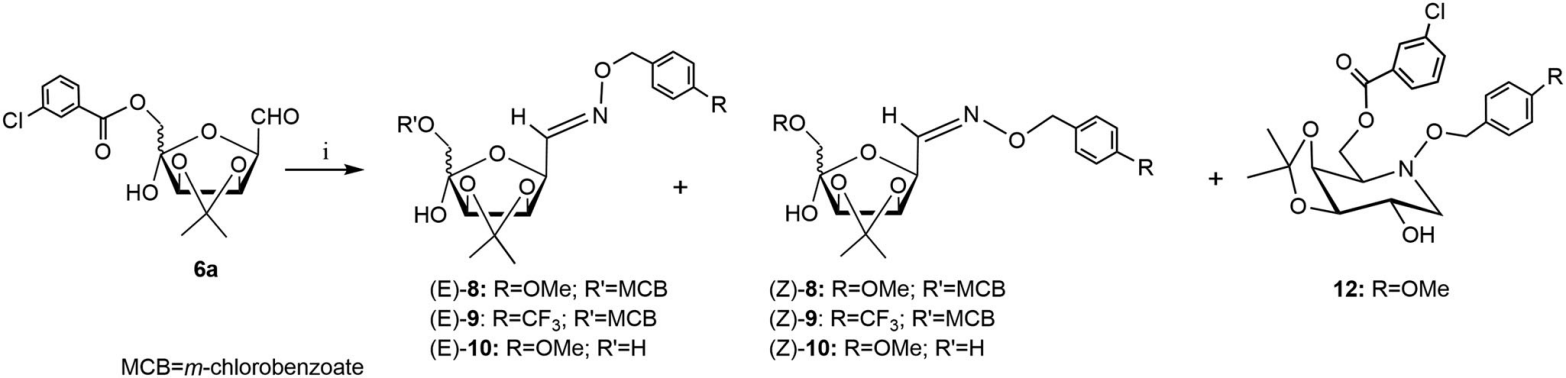
*Reagents and conditions*: (i) **7a**, NaBH_3_CN, MeOH, 60 °C, 96 h (**12**: 20%; *E/Z*-**8**: 10%; *E/Z*-**10**: 40%) or **7a**, NaBH_3_CN, MeOH, AcOH, 60 °C, 96 h, (**12**: 15%; *E/Z*-**8**: 12%; *E/Z*-**10**: 43%) or **7a**, NaBH_3_CN, MeOH, AcOH, MW irradiation, 40 °C, 30 min, (**12**: 25%; *E/Z*-**8**: 22%; *E/Z*-**10**: 11%).

Derivative **6a** was also reacted with *O*-(arylmethyl)hydroxylamine hydrochloride **7a** bearing an electron-donor group (methoxy group) (Scheme 3), in the condition of the intramolecular double reductive amination (aminocyclisation) following a protocol previously reported by us (NaBH_3_CN, MeOH, 60 °C, 96 h)^15^^,^^19^^,^^26^ or performed in presence of AcOH (pH 5–6)^27^ .In both cases the purification of the crude products by flash chromatography on silica gel afforded the pure azasugar **12** (d-*galacto*) in a rather low yield (15–20%), together with the mixture of *E/Z*-**8** oxime (10–12%) and the corresponding 6-*O*-deprotected *E/Z*-**10** isomers (40–43%).

The formation of similar products was also observed during the aminocyclisation of **6a** with **7a** under MW irradiation (NaBH_3_CN, MeOH, AcOH, pH 5–6, 40 °C, 30 min). Chromatographic separation on silica gel allowed the isolation of pure samples of **12**, (*E/Z*)-**8** oxime and (*E/Z*)-**10** in 25, 22 and 11% yield, respectively.

The aminocyclisation reaction (NaBH_3_CN, MeOH, AcOH, pH 5–6, 40 °C) of dicarbonyl derivative **6a** with **7 b** having an electron-withdrawing group (trifluoromethyl group) was performed under MW irradiation (Scheme 3). After 2 h the TLC analysis showed one spot together with several minor components. Purification by flash chromatography on silica gel of the crude product afforded only the corresponding arylmethyloxyimino derivative **9** (Scheme 2) as a mixture of (*E/Z*) isomers [(*E*)-**9**:(*Z*)-**9 **=** **60:40] in a 55% yield.

Reduction of the isomeric mixture (*E/Z*)-**8** with NaBH_3_CN in dry MeOH in the presence of AcOH (Scheme 3) was very slow (6 days) and the purification by flash chromatography on silica gel of crude product afforded pure azapyranose **12** (45%) and the starting material (*E/Z*)-**8** (25%). A similar result was obtained when the reduction of mixture (*E/Z*)-**8** was conducted in a microwave sealed tube (60 °C, 30 min). In this case, purification of crude product by flash chromatography on silica gel gave pure (*E/Z*)-**8** (35%) and azasugar **12** (33% yield). The isomeric mixtures (*E/Z*)-**9** were recovered unchanged after treatment with NaBH_3_CN in dry MeOH in presence of glacial AcOH (pH 5–6).

All new compounds (**6a** and **8–12**) were characterised and their mono- and two-dimensional NMR analyses (^1^H, ^13^C, DEPT-135, COSY, HSQC) were consistent with their structures (see Experimental Section and Supplementary Material). The (*Z)* and (*E*) forms of the oximes **8–11** were assessed by their NMR spectra (Tables 1 and 2) on the basis of the known effects caused by the oxime oxygen atom on the chemical shifts of cis vicinal carbons^28^ and protons^29^. By comparing the data reported in Tables 1 and 2, we can observe that the presence of the arylmethyloxyimino group at C-1 determines noticeable changes in the spectral parameters of the oximes both of the *E* and the *Z* series. In particular, H-1 and C-1 of the *Z* isomers are always shifted towards higher fields by about 0.66–0.61 ppm and 1.3–1.5 ppm respectively, with respect to the *E* isomers. Conversely, H-2 and C-2 signals of the *E* isomers are shifted towards higher fields by about 0.55–0.48 ppm and 2.1–1.2 ppm respectively with respect to the *Z* isomers (Tables 1 and 2).

**Table 2.** Selected ^13 ^C NMR data (δ, ppm) of d-*arabino*-aldohexos-5-ulose derivative **6a** and of arylmethyloxyimino derivatives **8–11**.

Compound	Solvent	C-1	C-2	C-3	C-4	C-5	C-6
**6a**	CDCl_3_	197.6	81.5	83.7	84.3	104.9	65.3
**(*E*)-8**	CD_3_CN	147.6	77.7	82.9	86.3	104.5	66.4
**(*Z*)-8**	CD_3_CN	149.0	75.6	81.8	85.7	104.6	66.4
**(*E*)-9**^a^	CD_3_CN	148.6	77.6	82.8	86.3	105.0	66.3
**(*Z*)-9**^a^	CD_3_CN	149.9	75.8	81.8	85.7	104.6	66.3
**(*E*)-10**^a^	CD_3_CN	149.5	77.2	82.9	86.0	106.1	63.9
**(*Z*)-10**^a^	CD_3_CN	148.0	75.1	81.8	85.5	105.7	63.9
**(*E*)-11**^a^	CD_3_CN	149.0	77.0	82.8	86.0	106.2	63.8
**(*Z*)-11**^a^	CD_3_CN	150.4	75.8	81.8	85.5	105.8	64.1

^a^ NMR data were obtained from analysis of isomeric mixture.

### 3.2. Biological activity

#### 3.2.1. Aldose reductase inhibitory assays

The novel arylmethyloxyimino derivatives **8–11** and the new azasugar **12** were tested against the human recombinant ALR2 in a cell-free assay. Results obtained are listed in Table 3.

**Table 3.** Aldose Reductase (ALR2) inhibitory data of compounds **8–11** and **12.**

Compound	% Inhibition of ALR2^a^**(100 μM)**
(*E*)-**8**	55
(*Z*)-**8**	83
(*E*)-**8**/(*Z*)-**8**=55:45	71
(*E*)-**9**/(*Z*)-**9**=93:7	52
(*E*)-**10**/(*Z*)-**10**=55:45	50
(*E*)-**11**/(*Z*)-**11**=60:40	56
**12**	10
**Tolrestat**	98

^a^ Percentage of enzyme inhibition at 100 μM test compound, obtained as mean of at least three determinations. Standard errors of the means (SEMs) are ≤10%.

All the compounds proved to inhibit the target enzyme when tested at 100 µM concentration, showing different degrees of efficacy. At first, both the arylmethyloxyimino derivatives **8** and **9** and the corresponding 6-*O*-deprotected compounds **10** and **11** were tested as isomeric (*E/Z*) mixtures, containing nearly equal amounts of the two forms. All but the (*E/Z*)-**8** sample halved the inhibitory activity of ALR2 notwithstanding the presence of the protecting *m*-chlorobenzoyl group, thus hinting that this lipophilic portion is not crucial for the structural recognition of the enzyme binding site. The higher activity displayed by the (*E/Z*)-**8** sample suggested that the two *E* and *Z* geometric forms might interact differently with the catalytic binding site of the enzyme. Actually, when tested in their isolated forms, the (*Z*)-isomer was more effective than the (*E*) parent. Clearly, in this form, the mutual geometric location of the polar furanose ring and the lipophilic aryloxy fragment fulfils better the structural requirements of the ALR2 binding site. This assumption is confirmed also by the functional datum obtained with the (*E/Z*)-**9** sample. Indeed, although tested as an isomeric mixture, the sample is mainly represented by the *E* isomer (93%, Table 3), and displayed an inhibitory activity fully comparable with the pure (*E*)-**8**. No significant activity was observed with the azasugar **12**.

#### 3.2.2. In vitro assessment of ALR2 inhibition

**Figure 4.**
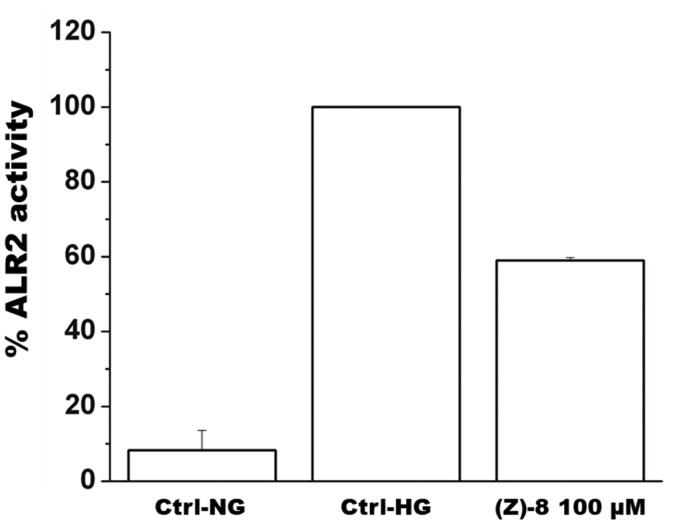
The histogram shows results obtained for the inhibition of ALR2 by using a specific kit assay after incubation with compound (*Z*)-**8**. Values are calculated as percentage of maximum activity measured in hyperglycaemic control Ctrl-HG) and are reported as mean ± SEM (*n* = 3, independent experiments). The values shown for the Ctrl-NG and Ctrl-HG correspond to those present in the previous work of the same authors^15^ because the molecules of the two works were evaluated in a single screening.

The geometric isomer (*Z*)-**8**, showing the best *in vitro* inhibitory activity against ALR2, was further investigated *in vitro* in a cell-based model on the murine retinoblastoma photoreceptor-like cell line 661w, endowed with ALR2 enzyme as previously demonstrated^15^. Figure 4 shows how compound (*Z*)-**8**, tested at 100 µM concentration, inhibited significantly ALR2 activity when cells were cultured in a high-glucose medium.

**Figure 5.**
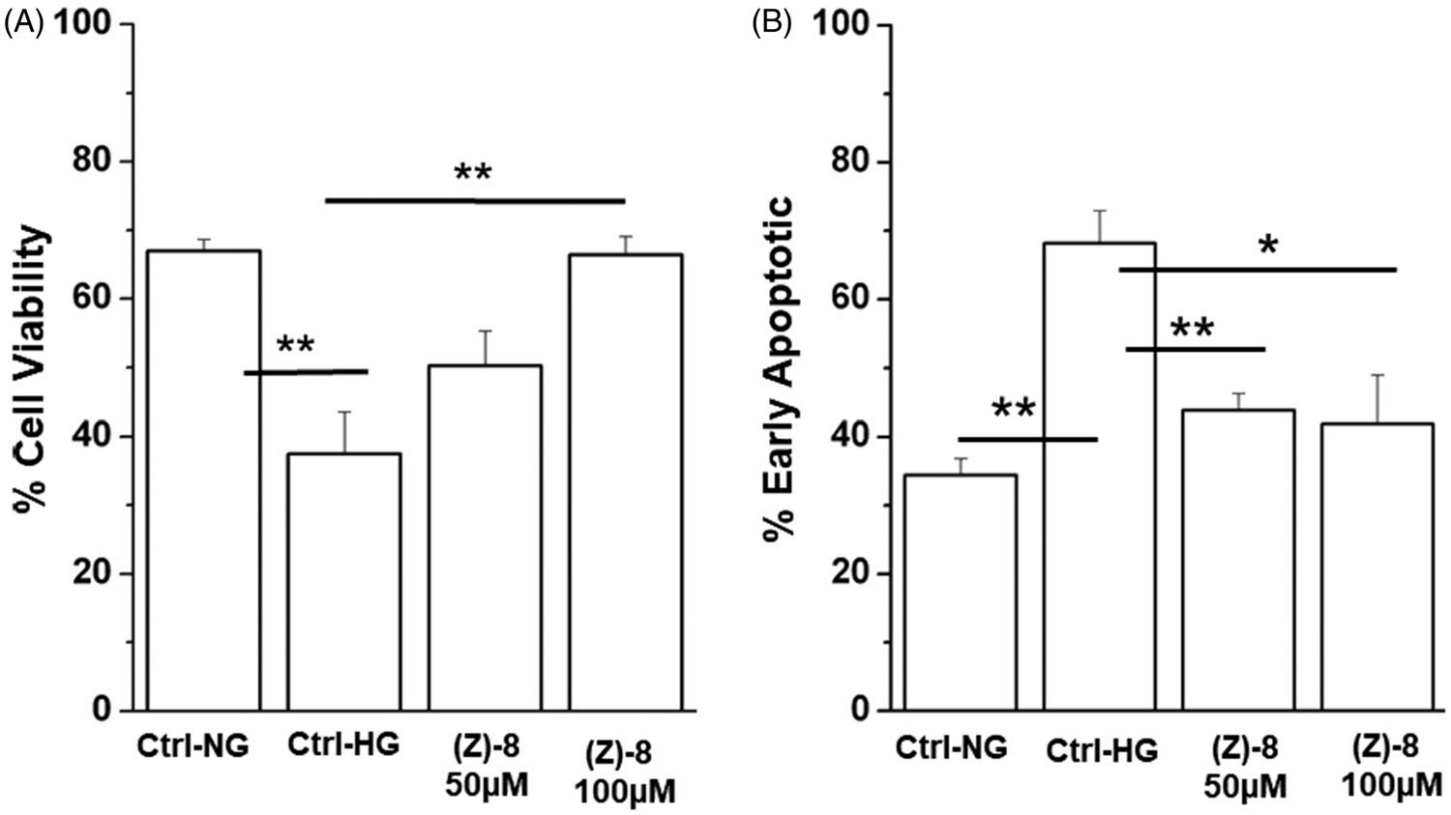
Effectiveness of compound (*Z*)-**8** in increasing cell viability. (A) The histogram shows that while the hyperglycaemic condition impairs significantly cell viability, compared to the normoglicemic control (Ctrl-NG), treatment with (*Z*)-**8** at both 50 and 100 µM concentrations recovers the level of cell viability, even significantly at 100 µM. (B) Histograms show results obtained for apoptotic pathway inhibition. It is important to note that the level of apoptotic process is significantly reduced after the treatment with both 50 and 100 µM of (*Z*)-**8**. Values were reported as mean ± SEM (*n* = 3, independent experiments); *t*-test *p < 0.05, **p < 0.01. The values shown for the Ctrl-NG and Ctrl-HG correspond to those present in the previous work of the same authors^15^ because the molecules of the two works were evaluated in a single screening.

Once demonstrated the ability of compound (*Z*)-**8** to inhibit the enzymatic activity of ALR2, the efficacy in increasing cell viability and reducing biological pathways triggering hyperglycaemia-induced cell death was also evaluated. Figure 5(A) shows how the treatment with two concentrations of (*Z*)-**8**, 50 and 100 µM, is able to increase the level of cellular vitality, even significantly for the highest dose (100 µM). Moreover, Figure 5(B) demonstrates the ability of the molecule under examination to reduce significantly the apoptotic process, one of the major pathways of death of retinal neurons, at both 50 and 100 µM concentrations.

**Figure 6.**
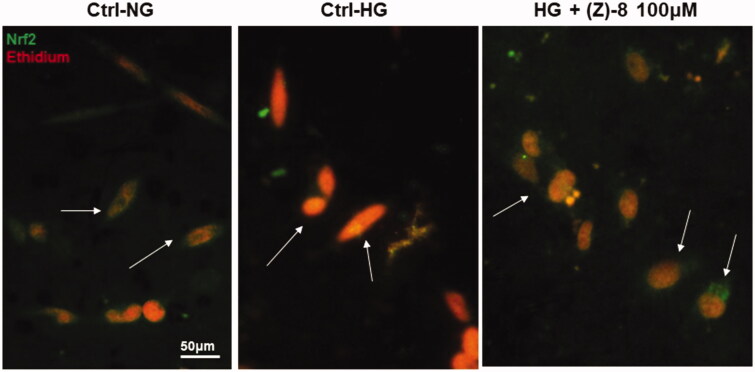
Antioxidant activity of compound (*Z*)-**8**. The micrograph obtained with the florescence microscope showed the specific staining for Nrf2 (green) and the nuclear staining obtained with ethidium bromide (red). The arrows indicate the different localisation of Nrf2, cytosolic in both normoglycemic conditions and after the treatment with 100 µM (*Z*)-**8**, while it is nuclear (yellow-orange marking, co-localisation) in hyperglycaemic conditions.

The ability of (*Z*)-**8** to counteract oxidative stress triggered by hyperglycaemic conditions was evaluated as well. Figure 6 shows the immunocytochemistry for Nrf2 as a transcription factor for phase 2 antioxidant enzymes. Under physiological conditions, Nrf2 is found in the cytosol complex with the inhibitory factor Keap1. In conditions of high oxidative stress, such as the hyperglycaemic ones, ROS induce the disruption of the Nrf2-Keap1 complex and while Keap1 is degraded, Nrf2 translocate to the nucleus where it interacts with specific DNA sequence called ARE (antioxidant response element) inducing the increase in the synthesis of antioxidant enzymes such as Sod1. As can be seen from Figure 6(A), in the Ctrl-NG the localisation of Nrf2 is cytosolic (green staining) and well separated from the red nuclear staining. Conversely, in the Ctrl-HG, the green cytosolic staining disappear and a yellow-orange colour of the nucleus becomes visible, indicating that Nrf2 translocation occurred in the nucleus in response to the increased ROS. Treatment with (*Z*)-**8** at 100 µM restored the localisation of cytosolic Nrf2, completely comparable to Ctrl-NG.

**Figure 7.**
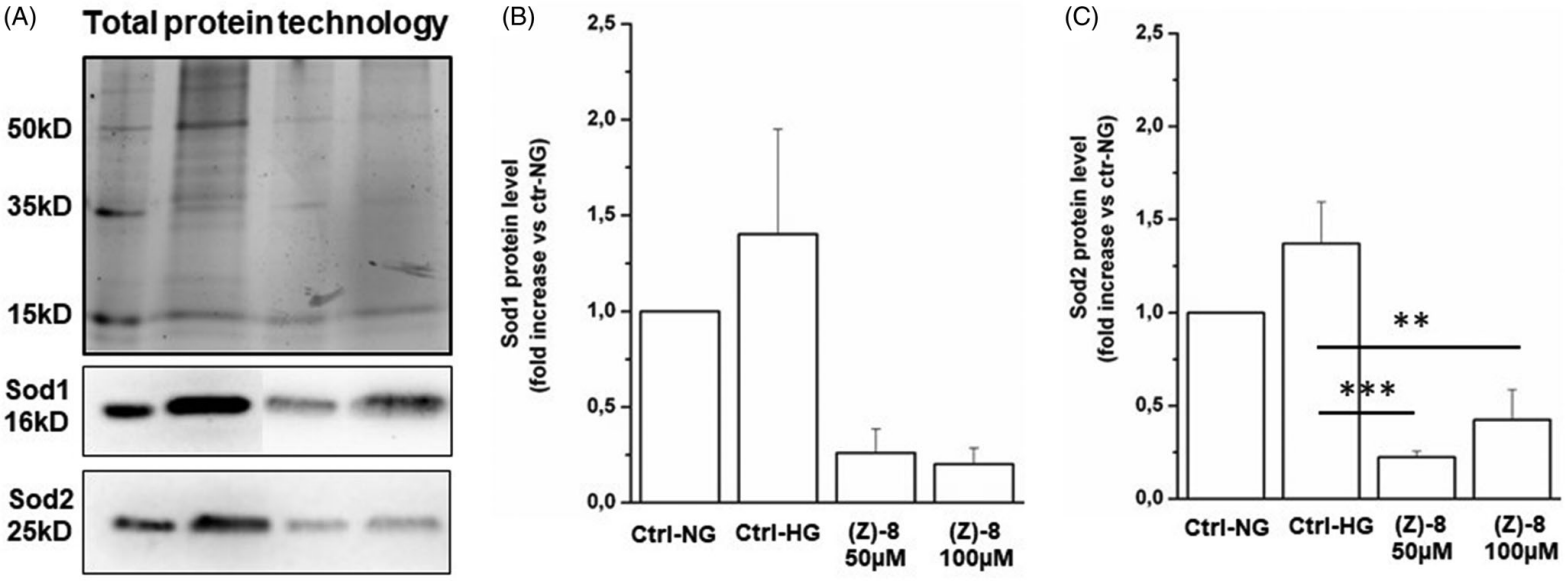
(A) Representative image of a western blotting experiment. Contents of Sod1 and Sod2 were normalised for total protein content by using the Stain Free Technology (BioRad). The graph bar shows the protein levels of the antioxidant enzyme Sod1. The treatment with (*Z*)-**8** is effective in decreasing protein levels in respect to either Ctrl-NG or Ctrl-HG, in a dose-dependent manner. (C) The graph bar shows the protein levels of the antioxidant enzyme Sod2. In this case, the treatment with (*Z*)-**8** is effective in significantly decreasing protein levels in respect to either Ctrl-NG or Ctrl-HG. Values are reported as mean ± SEM (*n* = 3, independent experiments); *t*-test **p < 0.01, ***p < 0.001. The values shown for the Ctrl-NG and Ctrl-HG correspond to those present in the previous work of the same authors^15^ because the molecules of the two works were evaluated in a single screening.

The result obtained with immunocytochemistry technique is also confirmed by data obtained by biochemical evaluation of Sod1 and Sod2 levels. Actually, as shown in Figure 7(B,C), 100 µM dose of (*Z*)-**8** is able to reduce both Sod1 and Sod2 levels, indicating a reduction of ROS levels due to the scavenger activity of the compound.

## 4. Conclusions

ALR2 plays a well-acknowledged crucial role in the onset and development of long term diabetic complications. Therefore, the obtainment of compounds able to counteract its activity represents a key and challenging research field. Pursuing our interest in the development of innovative ARIs, we described here a novel class of oxyimino derivatives, obtained by reaction of a 1,5-dicarbonyl substrate with *O*-(arylmethyl)hydroxylamines. Besides gaining knowledge on the reactivity of this kind of compound, our investigation succeeded in obtaining a novel prototypical class of effective ALR2 inhibitors. The 1,5-dicarbonyl derivative **6**, obtained from the 6-*deoxy*-hex-5-enol ether **1** through a conventional epoxidation reaction, proved to exist mainly in its furanose tautomeric form (**6a**), and reacted with suitably substituted *O*-(arylmethyl)hydroxylamines to afford the corresponding oxime derivatives as *E/Z* isomeric mixtures. These latter proved to last through any reductive attempts, providing the *O*-deprotected derivatives instead of aryloxyamino compounds whatever the experimental conditions applied. Although unexpected, the novel oxyimino derivatives turned out to be intriguing functional chemotypes. Indeed, they all proved to inhibit the target ALR2, thus demonstrating key structural elements to bind the catalytic site of the enzyme. Moreover, compound (*Z*)-**8**, showing the best ALR2 inhibitory activity, reduced both cell death and the apoptotic process of photoreceptor-like 661w cell line exposed to high-glucose medium, counteracting significantly the oxidative stress triggered by hyperglycaemic conditions. Results obtained, although preliminary, demonstrate that suitably substituted sugar analogues can provide in principle innovative ARIs, thus opening up a novel research opportunity to offer an effective therapeutic solution to diabetic population, which is expected to increase substantially in the coming years.

## Supplementary Material

Supplemental Material
