# Supplementary material for: Oxy-imino saccharidic derivatives as a new structural class of aldose reductase inhibitors endowed with anti-oxidant activity
Source: J Enzyme Inhib Med Chem. 2020 May 12;35(1):1194–205. doi: 10.1080/14756366.2020.1763331 (PMC7269086; doi:10.1080/14756366.2020.1763331)

Oxy-Imino Saccharidic Derivatives as a New Structural Class of Aldose Reductase Inhibitors Endowed with Anti-oxidant Activity

Felicia D'Andrea,[a*] Stefania Sartini,[a] Ilaria Piano,[a] Matteo Franceschi,[a] Luca Quattrini,[a] Lorenzo Guazzelli,[a] Lidia Ciccone,[a] Elisabetta Orlandini,[b,c] Claudia Gargini,[a] Concettina La Motta,[a*] Susanna Nencetti.[a*]

[a]*Department of Pharmacy, University of Pisa, Via Bonanno 6/33, 56126, Pisa, Italy.* [b]*Department of Earth Sciences, University of Pisa, Via Santa Maria 53, 56126, Pisa, Italy.* [c]*Research Center "E. Piaggio," University of Pisa, Pisa, 56122, Italy;*

Corresponding Authors:

Felicia D'Andrea. felicia.dandrea@unipi.it

Concettina La Motta concettina.lamotta@unipi.it

Susanna Nencetti. susanna.nencetti@unipi.it

Table of Contents

Biological assays

661w cell culture S3

In vitro ALR2 enzymatic inhibition. S3

Analysis of cellular viability and detection of apoptosis. S3

Immunocytochemistry. S3

Western blot. S4

References S4

NMR spectra of total new synthesized compounds

^{1}H, ^{13}C NMR, COSY and HSQC spectra of compound **1** S5-S6

^{1}H, ^{13}C NMR, DEPT-135, HSQC spectra of mixture two C-5 anomeric **6a** S7-S8

^{1}H and ^{13}C NMR spectra of mixture (E)-**8**+(Z)-**8**, of (E)-**8**, and (Z)-**8** S9

^{1}H, ^{13}C NMR, COSY and HSQC spectra of (E)**-8** S10-S11

^{1}H, ^{13}C NMR, COSY and HSQC spectra of (Z)**-8** S12-S13

^{1}H, ^{13}C NMR, COSY and HSQC spectra of mixture of (E)**-9**+(Z)**-9** S14-S15

^{1}H, ^{13}C NMR, and DEPT-135 spectra of mixture of (E)**-10**+(Z)**-10** S16-S17

^{1}H, ^{13}C NMR, DEPT-135 and COSY spectra of mixture of (E)**-11**+(Z)**-11** S18-S19

^{1}H, ^{13}C NMR, DEPT-135, COSY and HSQC spectra of compound **12** S20-S21

Biological assays

The biological assays were performed following a previously described protocol[1]

661w cell culture.

The 661W cell line, derived from immortalized cone photoreceptors (provided by Muayyad Al-Ubaidi, University of Oklahoma), was maintained in Dulbecco's modified Eagle's medium (DMEM, Invitrogen, ThermoFisher Scientific, Waltham, MA) containing 4.5 g/l (24.5 mM) glucose and supplemented with 10% (v/v) heat-inactivated FBS, 1% L-glutammine and 1% pen/strep. For the experiments, confluent cells were maintained in 24.5 mM glucose (normoglycemic condition) or cultured in 55 mM glucose (hyperglycemic condition) during 24 h in the presence or absence of the test compound (*Z*)-**8**.

In vitro ALR2 enzymatic inhibition.

Briefly, cells were seeded in 24-well tissue culture plates and allowed to stabilize overnight in a 5% CO_2 incubator at 37 °C. Then, they were cultured in 24.5 mM (NG) or 55 mM (HG) glucose-containing medium in the absence or presence of (*Z*)-**8** (50 and 100 μM) for 24 h. After that, cells were mechanically lysate and the supernatant were collected by centrifugation and incubated with Aldo-Keto Reductase (AKR) Activity Assay Kit (AbCam) in a 96-well for 2 h, then absorbance was measured with a spectrophotometer (NanoQuant) at 450 nm.

Analysis of cellular viability and detection of apoptosis.

Briefly, quantification of cellular viability and apoptosis were performed using flow cytometry methods. Cells were seeded in 24-well tissue culture plates and allowed to stabilize overnight in a 5% CO_2 incubator at 37 °C. Then, they were cultured in 24.5 mM (NG) or 55 mM (HG) glucose-containing medium in the absence or presence of (*Z*)-**8** (50 and 100 μM) for 24 h. After the induction of HG condition, adherent and non-adherent cells were collected by centrifugation and incubated with Guava® ViaCount® Reagent or Muse™ Annexin V & Dead Cell Kit (Millipore) which, thanks to the presence of specific DNA-binding dyes, are able to distinguish viable, apoptotic, and dead cells. Data collected were analyzed with Muse 1.5 Software (Millipore).

Immunocytochemistry.

Briefly, cells were directly washed with PBS and fixed in PAF for 1 min, then washed with PBS twice for 10 min and incubated with PBS containing 0.03% Triton and 5% of bovine serum

albumin (1 h at room temperature), and the primary antibody anti-Nrf2 (1:50, overnight at 4°C). Samples were then washed three times with PBS and subsequently incubated with the secondary goat anti-rabbit immunoglobulin G (IgG; 1:1000; Vector Laboratories) 488-Alexa flour conjugated for 2 h at room temperature. After washing with PBS, samples were incubated with Ethidium Bromide (1:5000, Sigma-Aldrich) for 5 min. Finally, after washing twice with PBS, the chambers were detached from the slide and mounted with Vectashield mounting medium (Vector Laboratories). This immunolabeling protocol was modified respect to Wang et al., 2019.[2] The slides were examined with a fluorescent microscope (Nikon E-Ri). Images were processed with ImageJ software.

Western blot.

Levels of Sod1 and Sod2 proteins were normalized by the levels of their corresponding total proteins by using the Stain Free Technology (BioRad).[3] The chemiluminescence was analyzed by using the Chemidoc Quantified (Bio-Rad) and the images obtained analyzed with ImageLAb Software, with the normalization of protein levels versus the total protein content (Bio-Rad Techonologies).

References

[1] Guazzelli L, D'Andrea F, Sartini S, Giorgelli F, Confini G, Quattrini L, Piano I, Nencetti S, Orlandini E, Gargini C, La Motta C. Synthesis and investigation of polyhydroxylated pyrrolidine derivatives as novel chemotypes showing dual activity as glucosidase and aldose reductase inhibitors. Bioorg Chem 2019; 92:103298. doi: 10.1016/j.bioorg.2019.103298

[2] Wang J, Zhao J, Cui X, Mysona BA, Navneet S, Saul A, Ahuj M, Lambert N, Gazaryan IG, Thomas B, Bollinger EK, Smitha SB. The molecular chaperone sigma 1 receptor mediates rescue of retinal cone photoreceptor cells via modulation of NRF2. Free Radical Biology and Medicine 2019; 134:604-616. doi: 10.1016/j.freeradbiomed.2019.02.001.

[3] Gürtler A, Kunz N, Gomolka M, Hornhardt S, Friedl AA, McDonald K, Kohn JE, Posch A. Stain-Free technology as a normalization tool in Western blot analysis. Anal Biochem 2013; 433:105-11. doi: 10.1016/j.ab.2012.10.010.

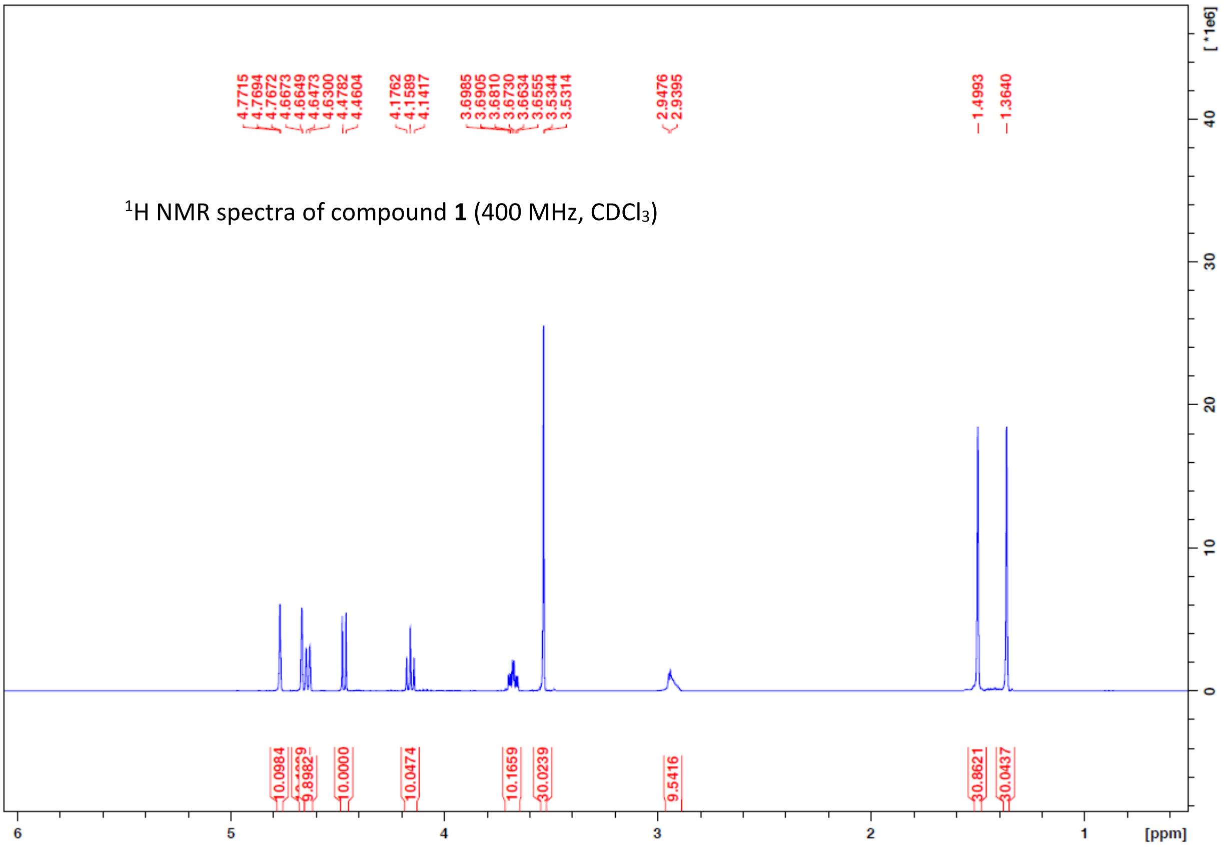

^{1}H NMR spectra of compound **1** (400 MHz, $CDCl_3$)

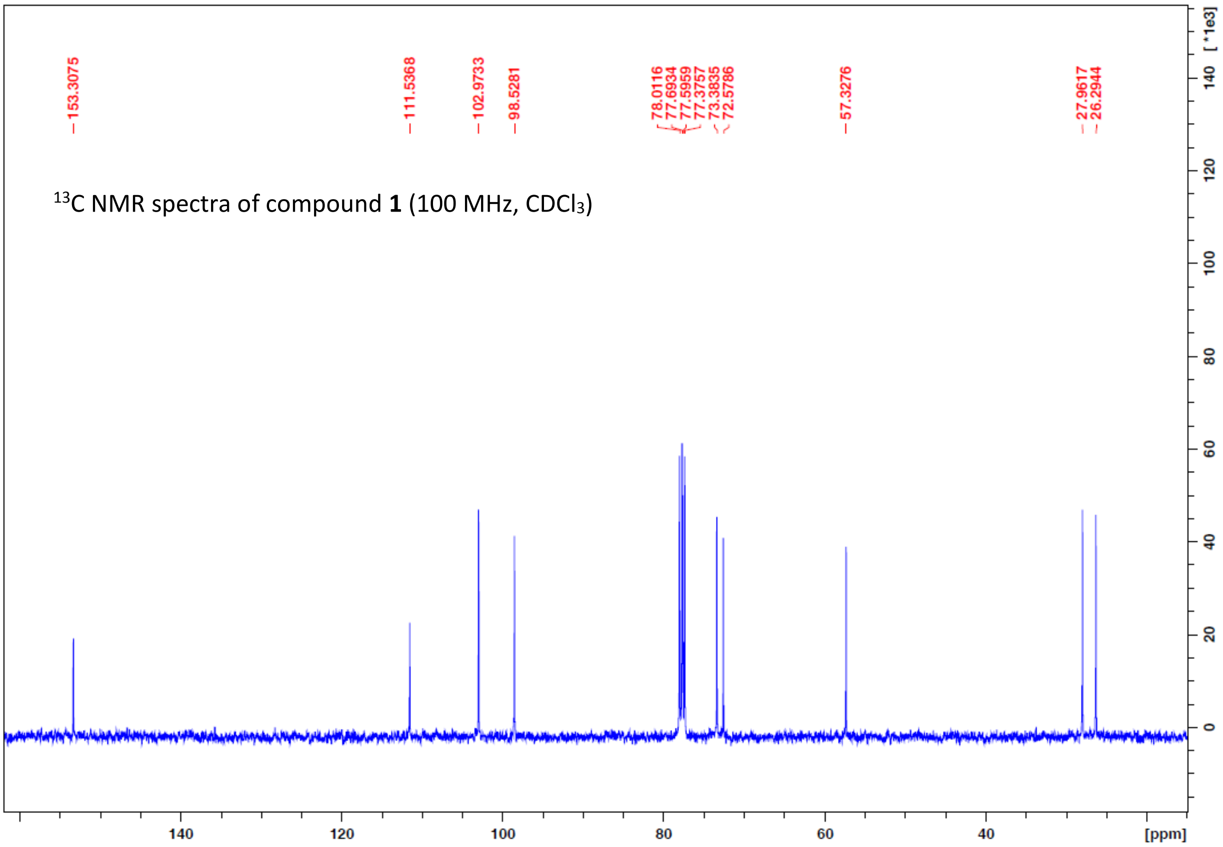

^{13}C NMR spectra of compound **1** (100 MHz, $CDCl_3$)

COSY spectra of compound **1** (400 MHz, $CDCl_3$)

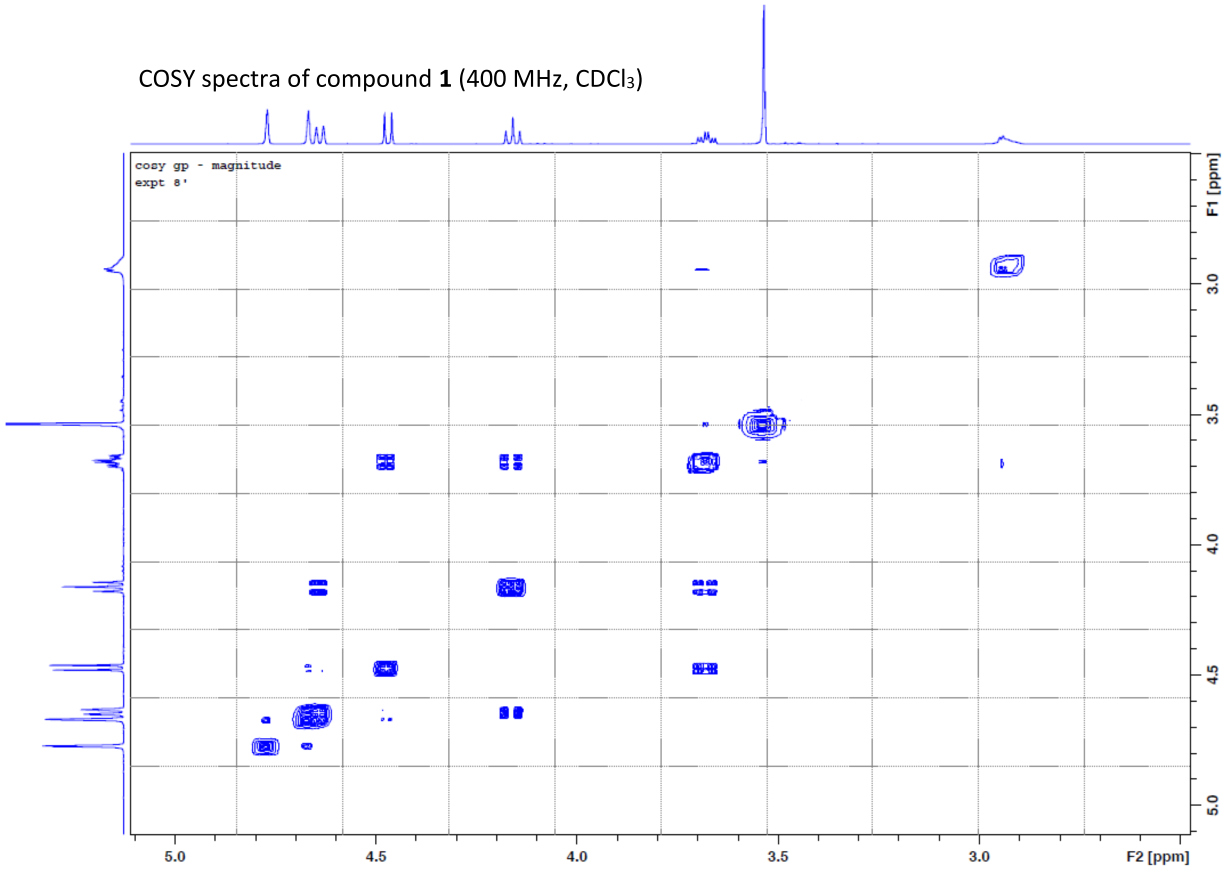

HSQC spectra of compound **1** (400 MHz, $CDCl_3$)

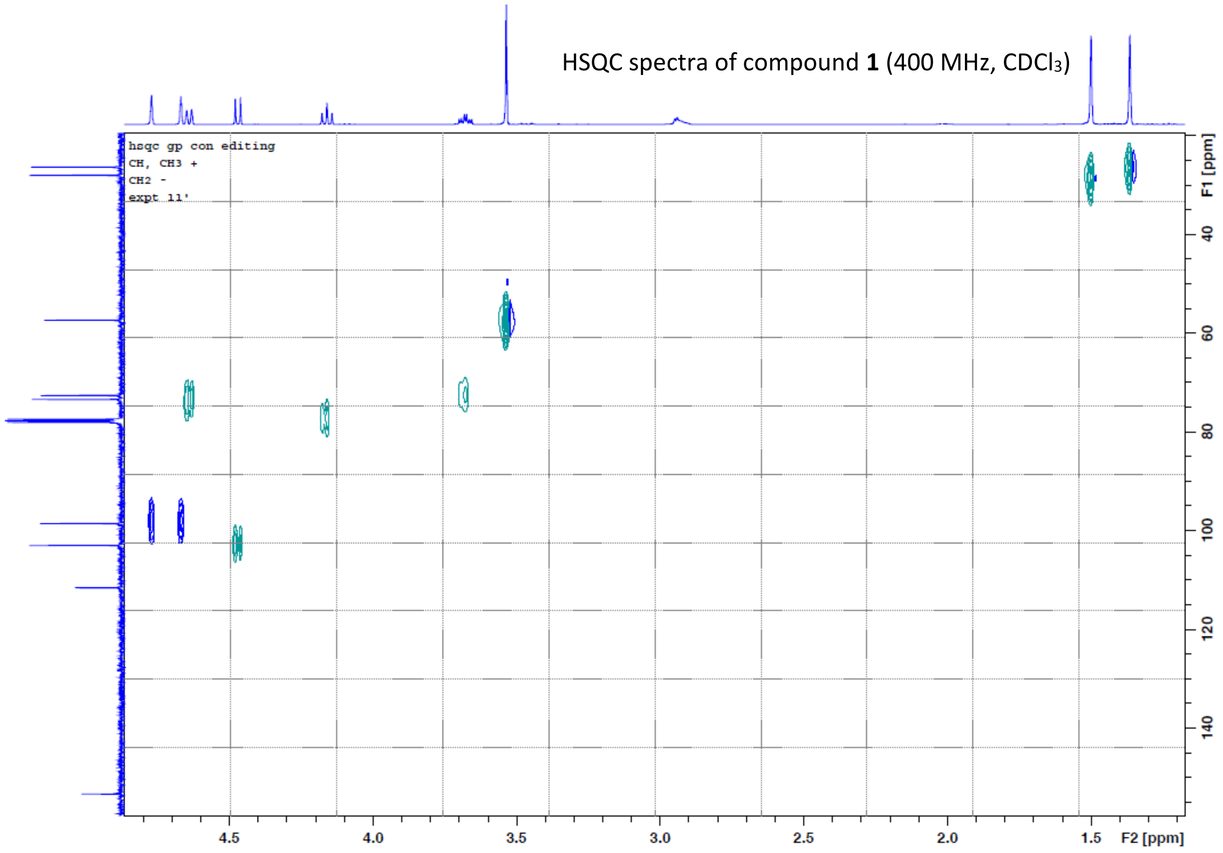

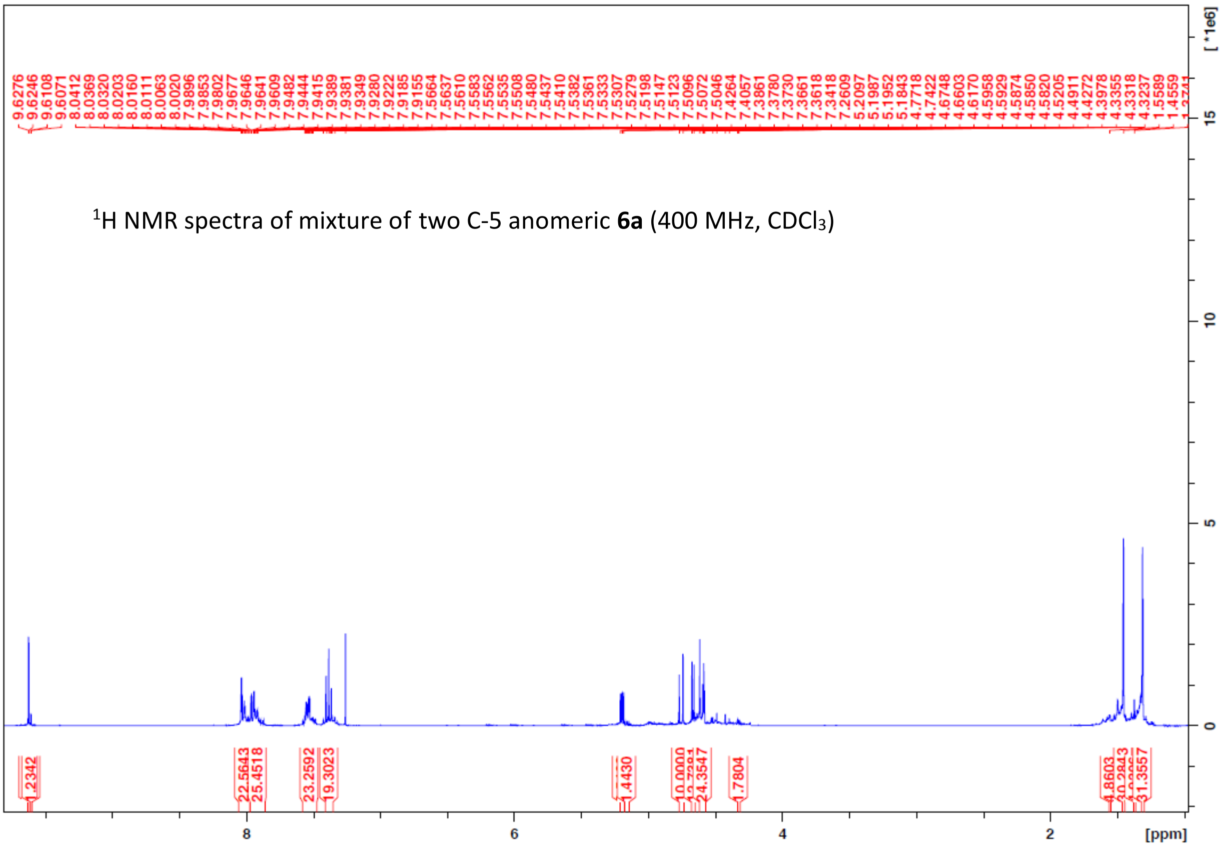

^{1}H NMR spectra of mixture of two C-5 anomeric **6a** (400 MHz, $CDCl_3$)

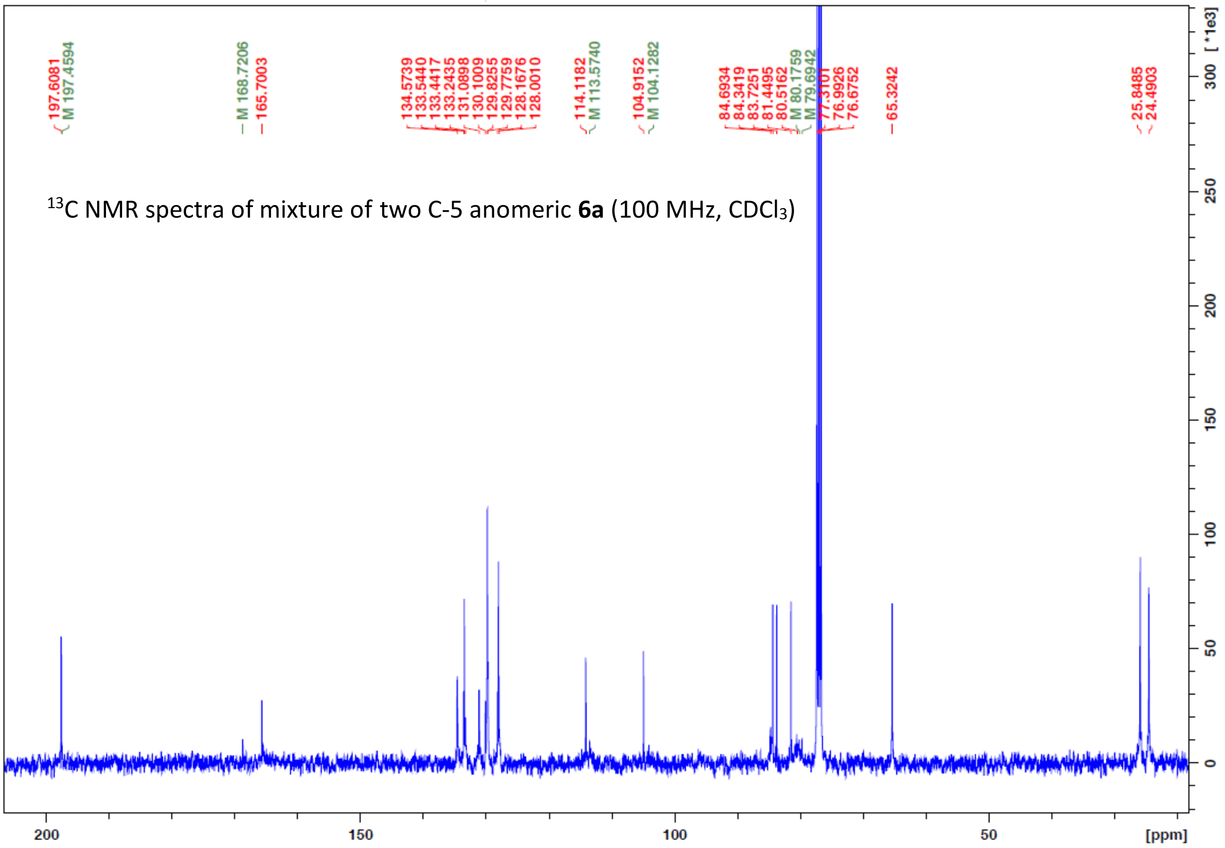

^{13}C NMR spectra of mixture of two C-5 anomeric **6a** (100 MHz, $CDCl_3$)

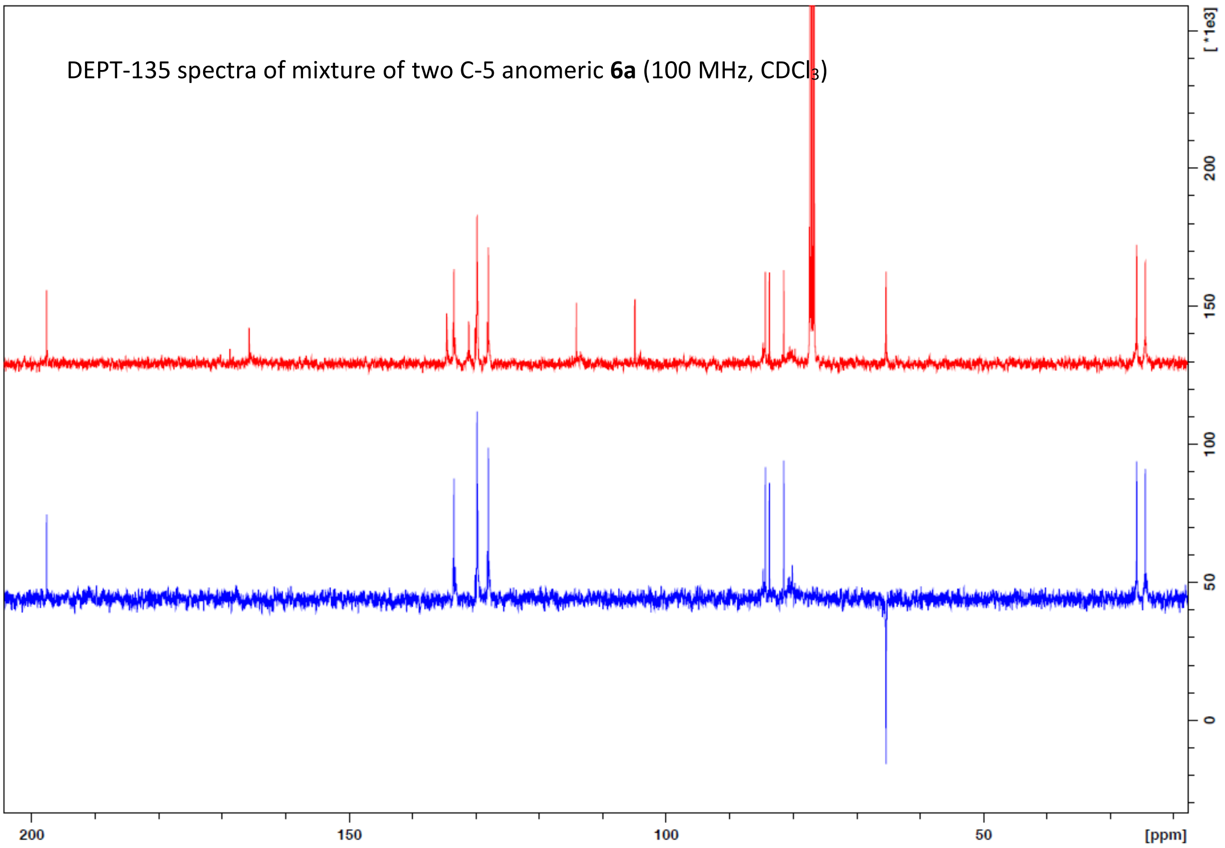

DEPT-135 spectra of mixture of two C-5 anomeric **6a** (100 MHz, $CDCl_3$)

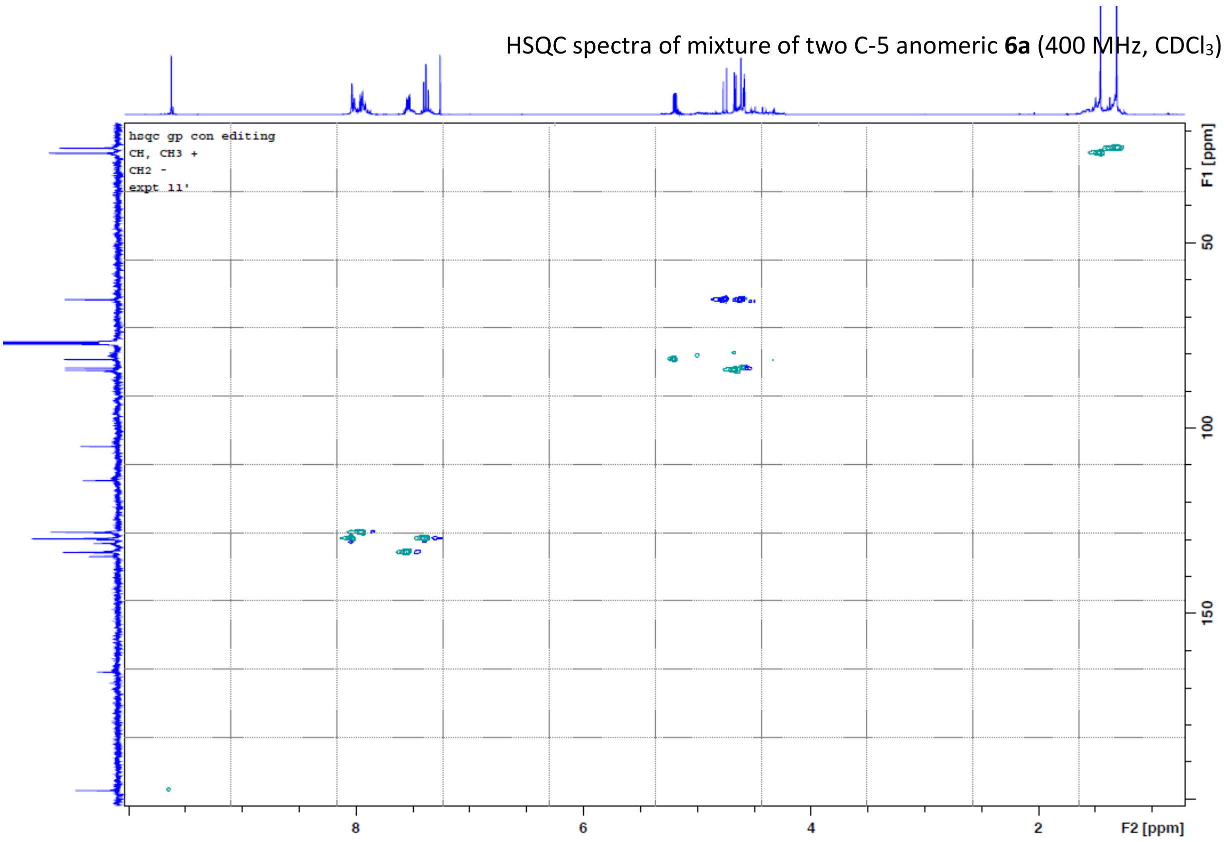

HSQC spectra of mixture of two C-5 anomeric **6a** (400 MHz, $CDCl_3$)

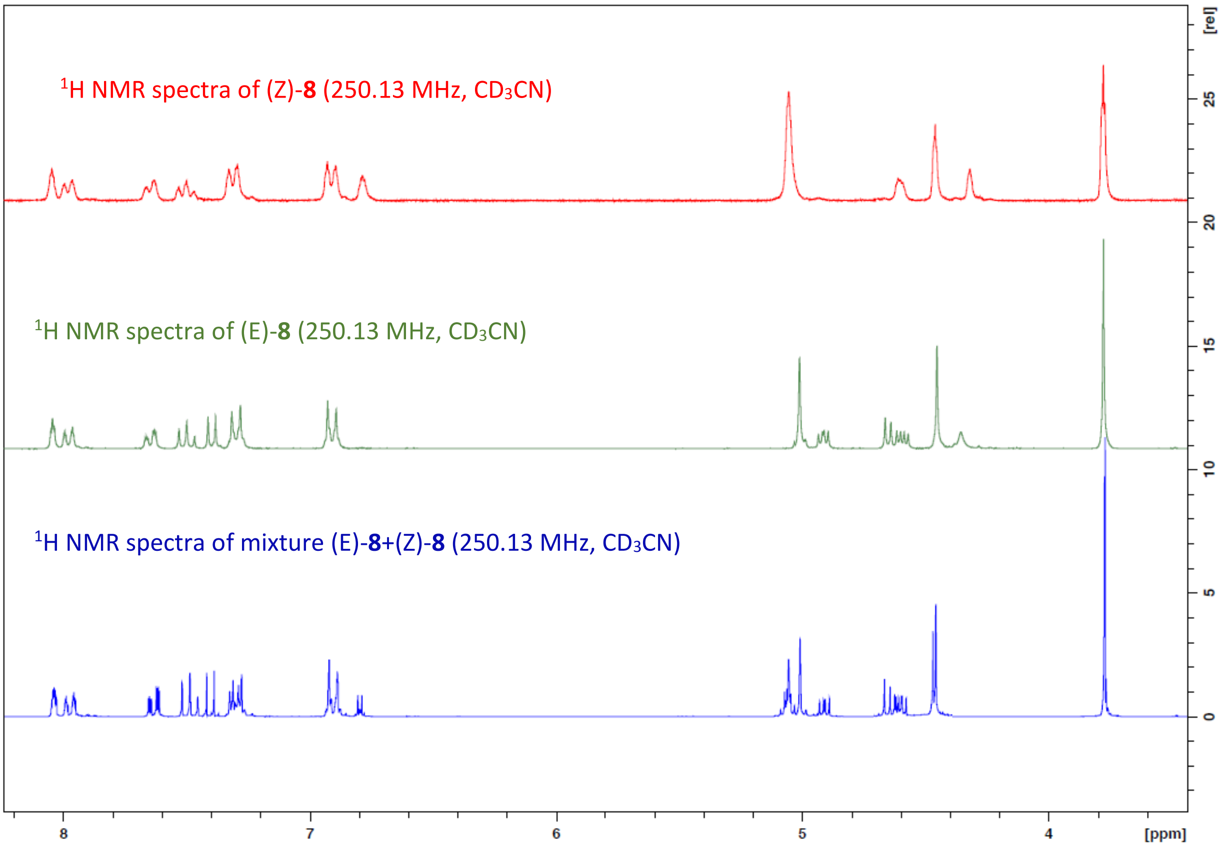

1H NMR spectra of (Z)-8 (250.13 MHz, CD3CN)
1H NMR spectra of (E)-8 (250.13 MHz, CD3CN)
1H NMR spectra of mixture (E)-8+(Z)-8 (250.13 MHz, CD3CN)
[rel]
[ppm]

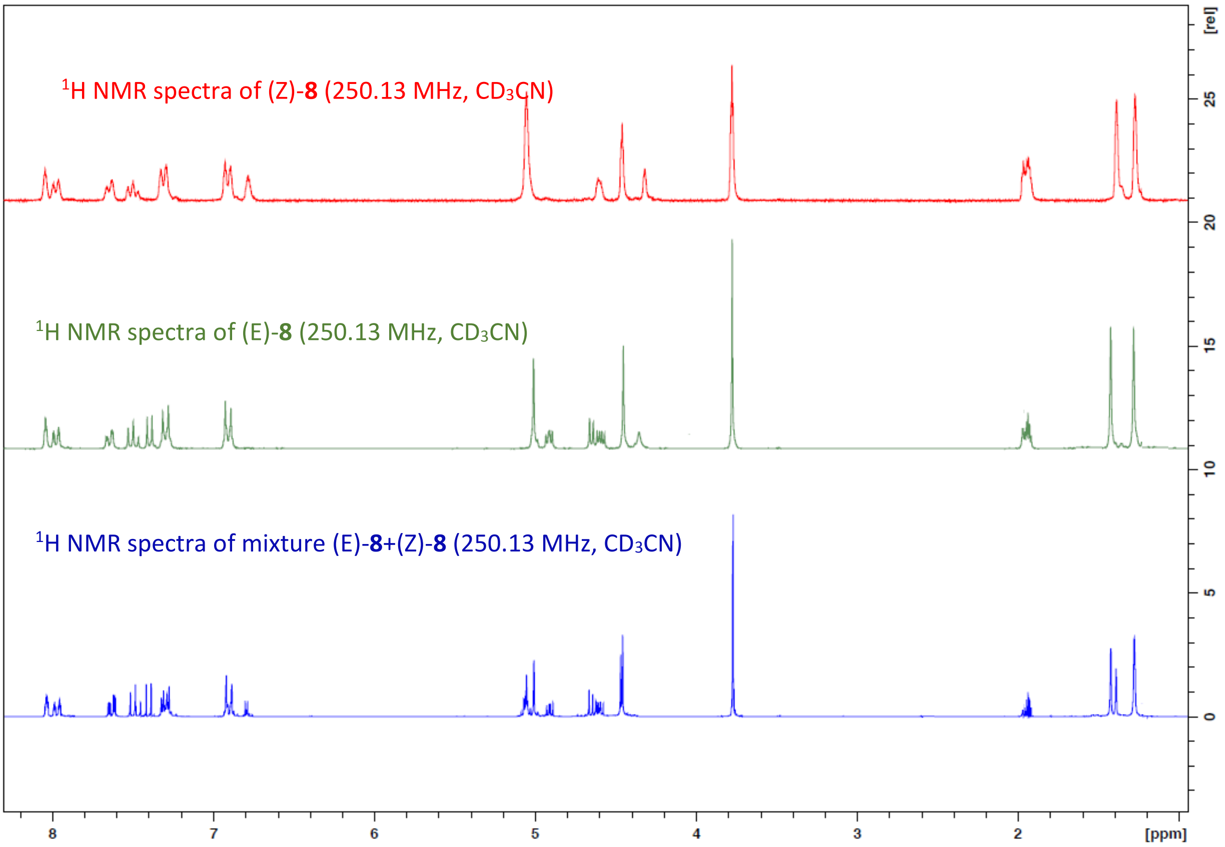

1H NMR spectra of (Z)-8 (250.13 MHz, CD3CN)
1H NMR spectra of (E)-8 (250.13 MHz, CD3CN)
1H NMR spectra of mixture (E)-8+(Z)-8 (250.13 MHz, CD3CN)
[rel]
[ppm]

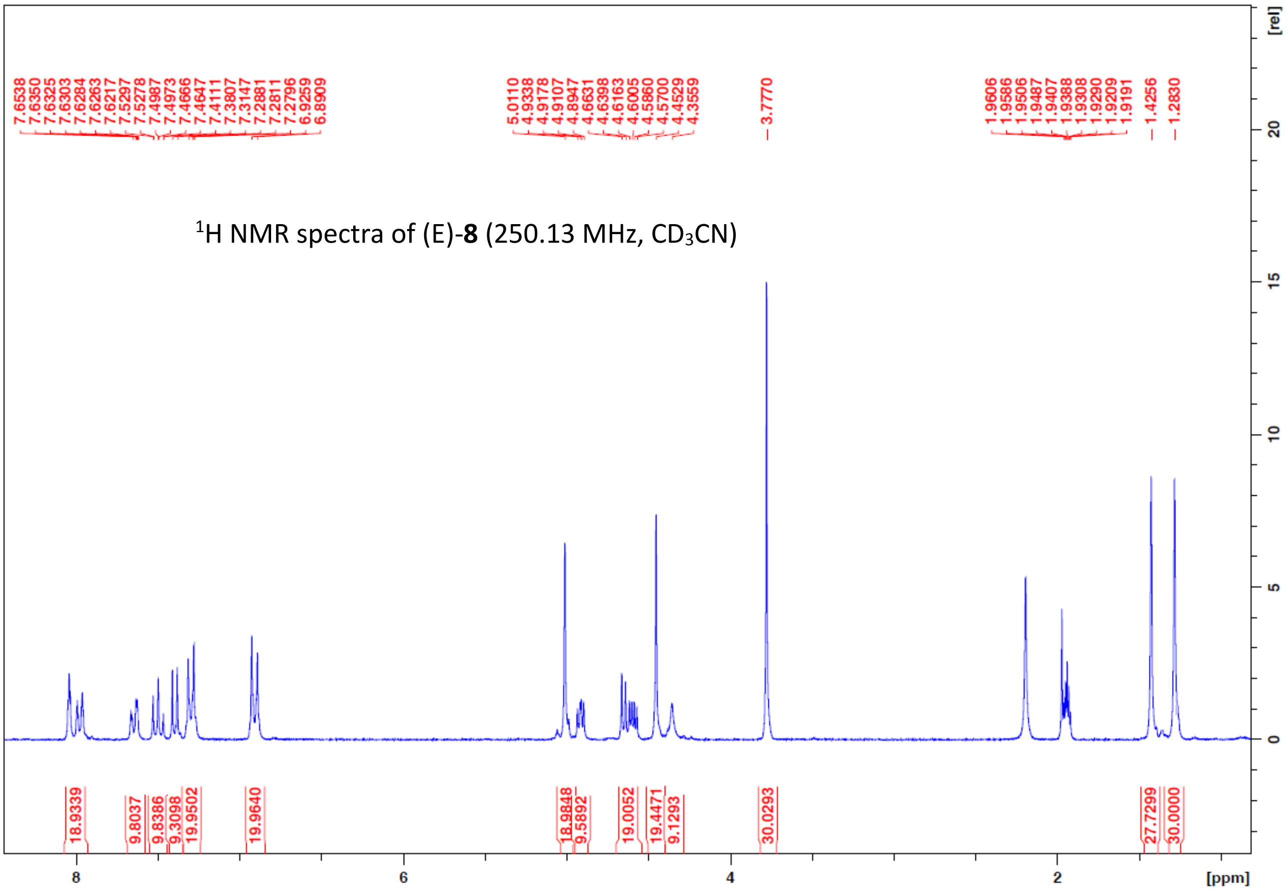

^{1}H NMR spectra of (E)-**8** (250.13 MHz, CD_3CN)

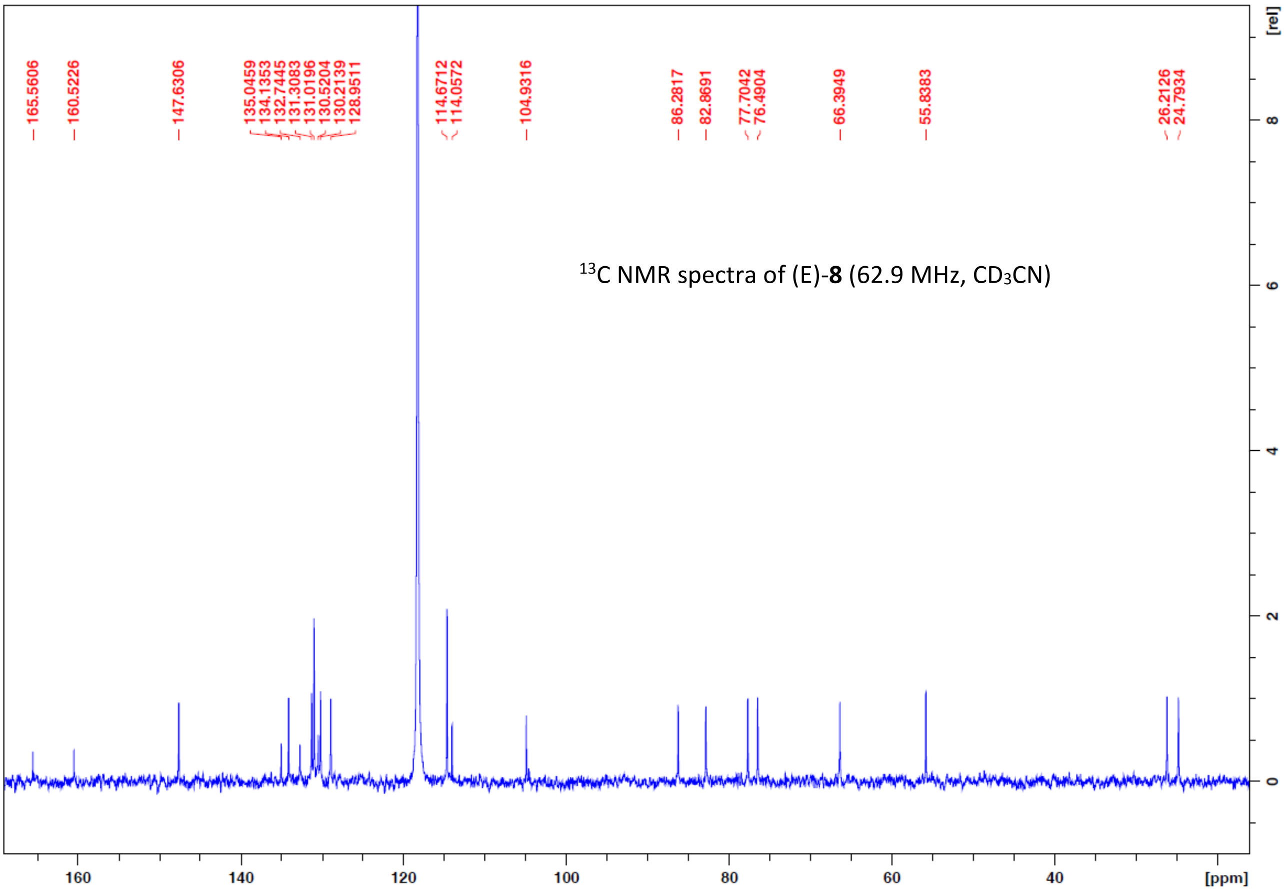

^{13}C NMR spectra of (E)-**8** (62.9 MHz, CD_3CN)

COSY spectra of (E)-**8** (250.13 MHz, CD_3CN)

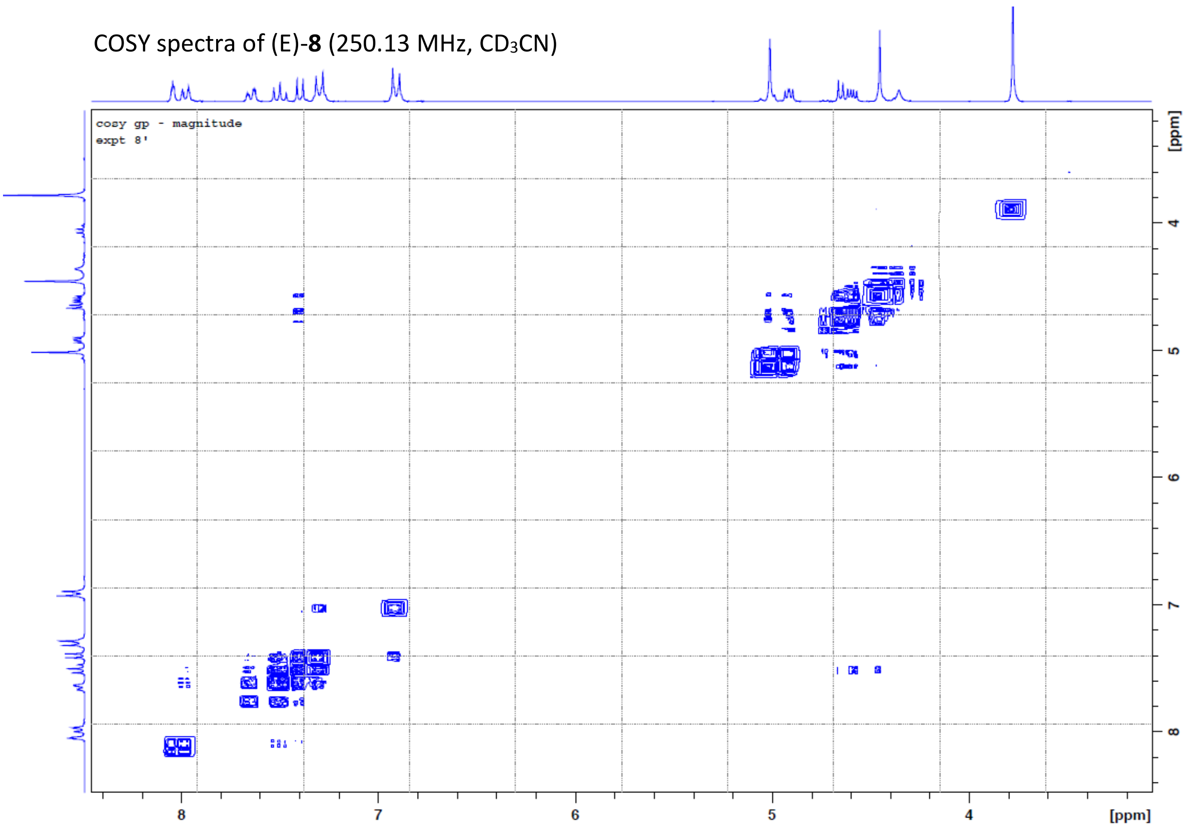

HSQC spectra of (E)-**8** (250.13 MHz, CD_3CN)

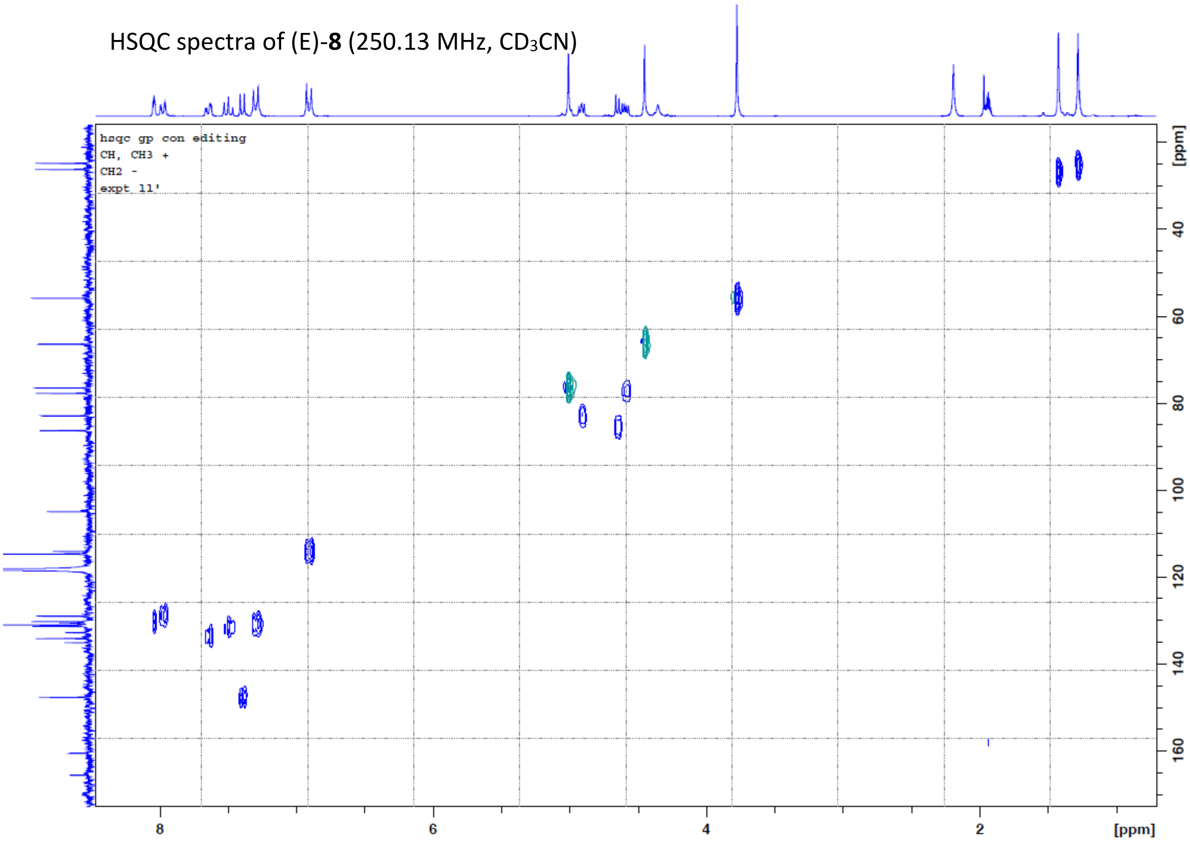

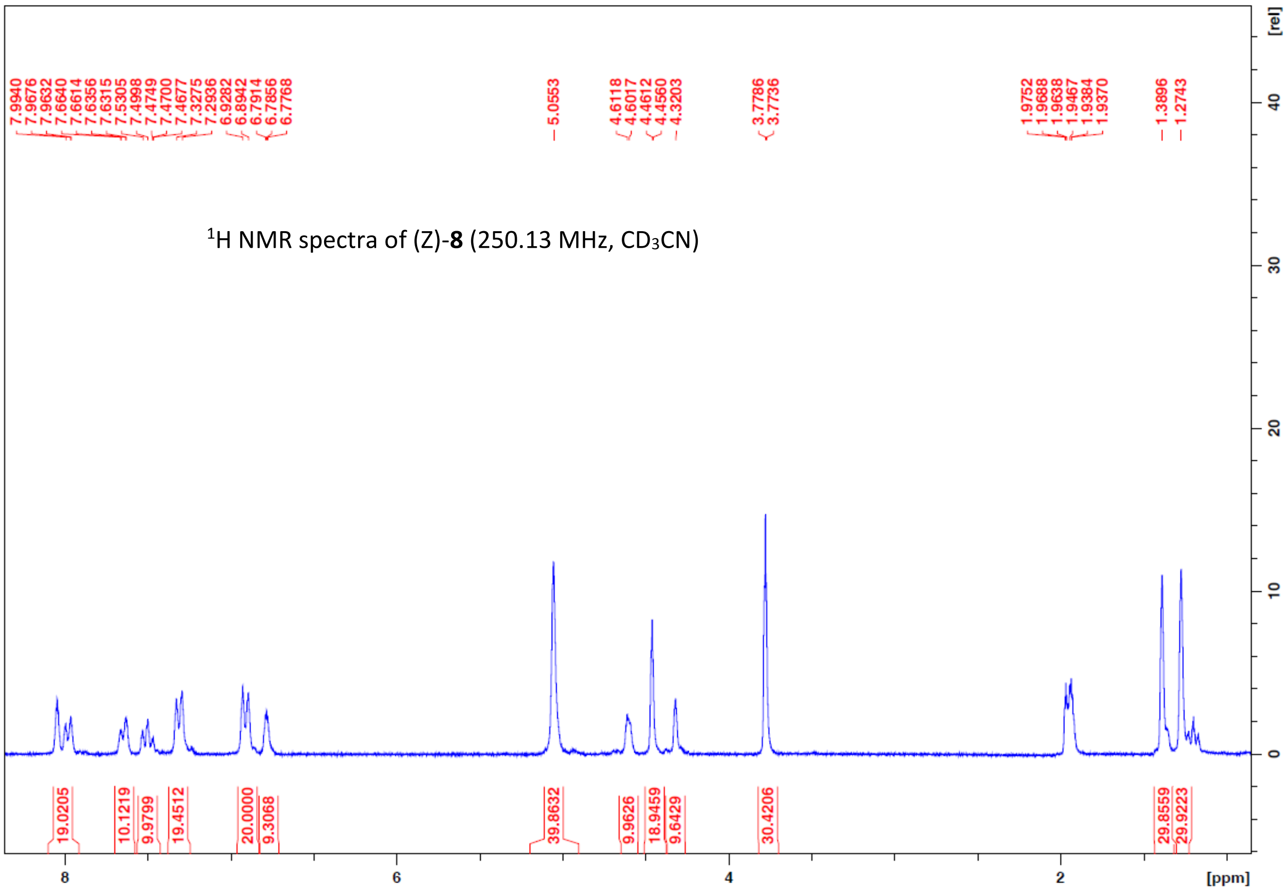

¹H NMR spectra of (Z)-8 (250.13 MHz, CD₃CN)
7.9940 7.9676 7.9632 7.6640 7.6614 7.6356 7.6315 7.5305 7.4998 7.4749 7.4700 7.4677 7.3275 7.2936 6.9282 6.8942 6.7914 6.7856 6.7768
5.0553
4.6118 4.6017 4.4612 4.4560 4.3203
3.7786 3.7736
1.9752 1.9688 1.9638 1.9467 1.9384 1.9370
1.3896 1.2743
19.0205 10.1219 9.9799 19.4512 20.0000 9.3068 39.8632 9.9626 18.9459 9.6429 30.4206 29.8559 29.9223
[rel]
[ppm]

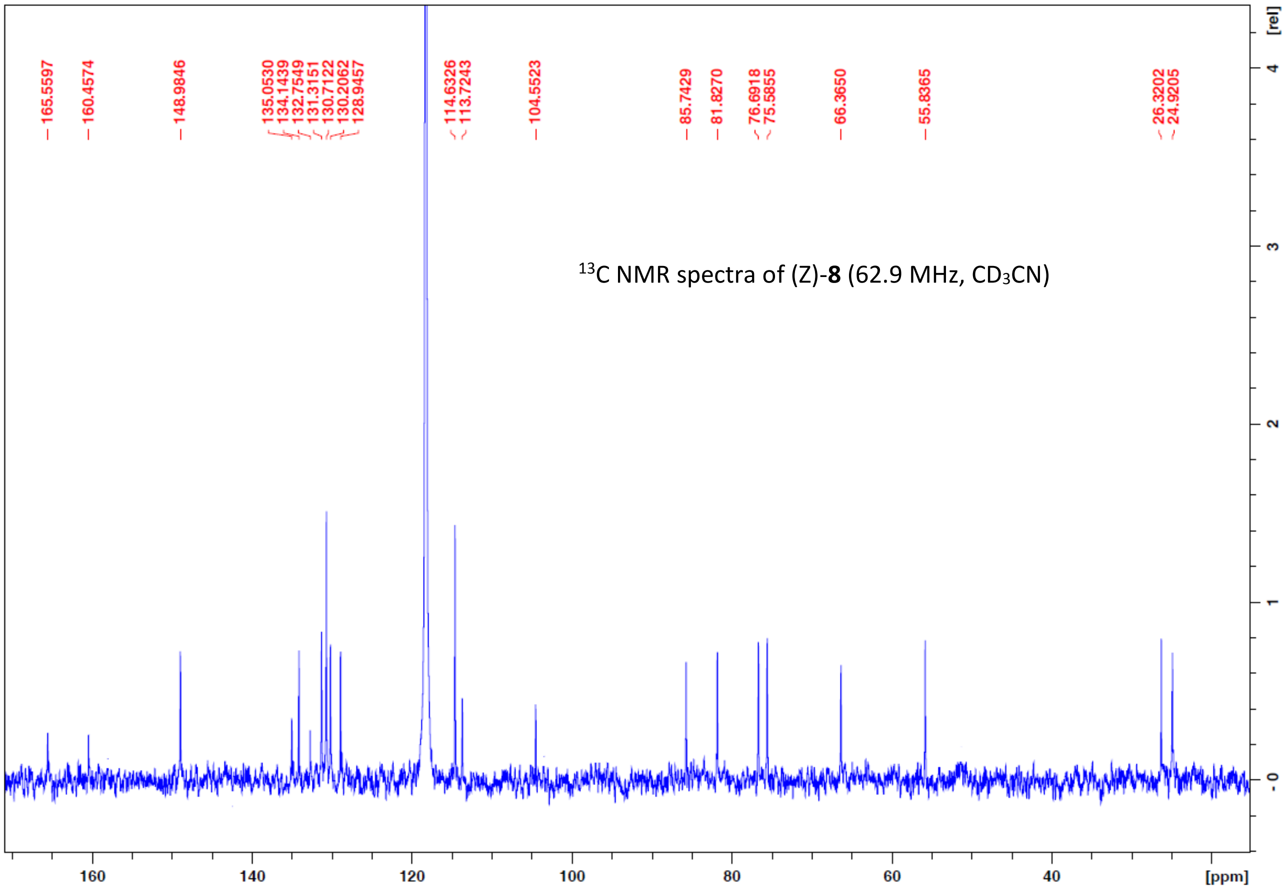

¹³C NMR spectra of (Z)-8 (62.9 MHz, CD₃CN)
165.5597 160.4574 148.9846 135.0530 134.1439 132.7549 131.3151 130.7122 130.2062 128.9457 114.6326 113.7243 104.5523 85.7429 81.8270 76.6918 75.5855 66.3650 55.8365 26.3202 24.9205
[rel]
[ppm]

COSY spectra of (E)-**8** (250.13 MHz, CD_3CN)

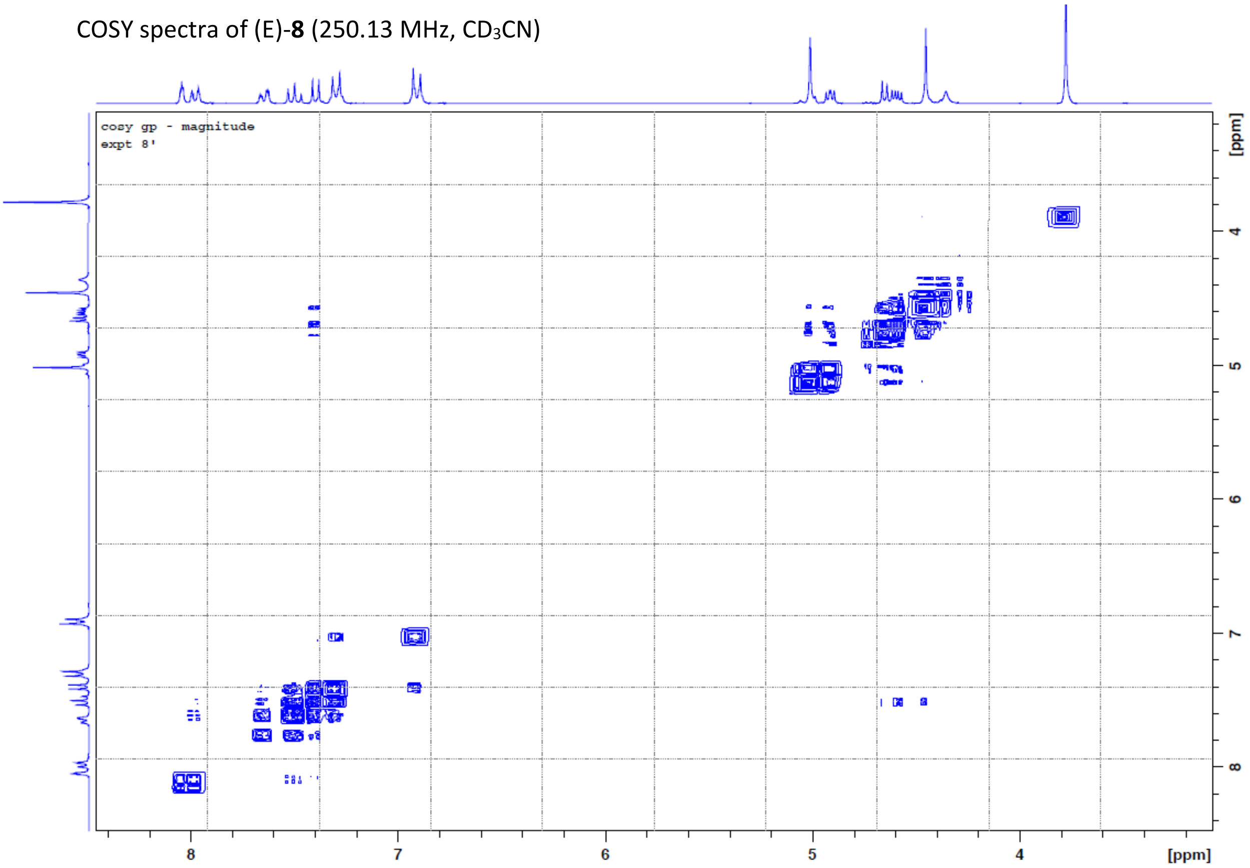

HSQC spectra of (Z)-**8** (250.13 MHz, CD_3CN)

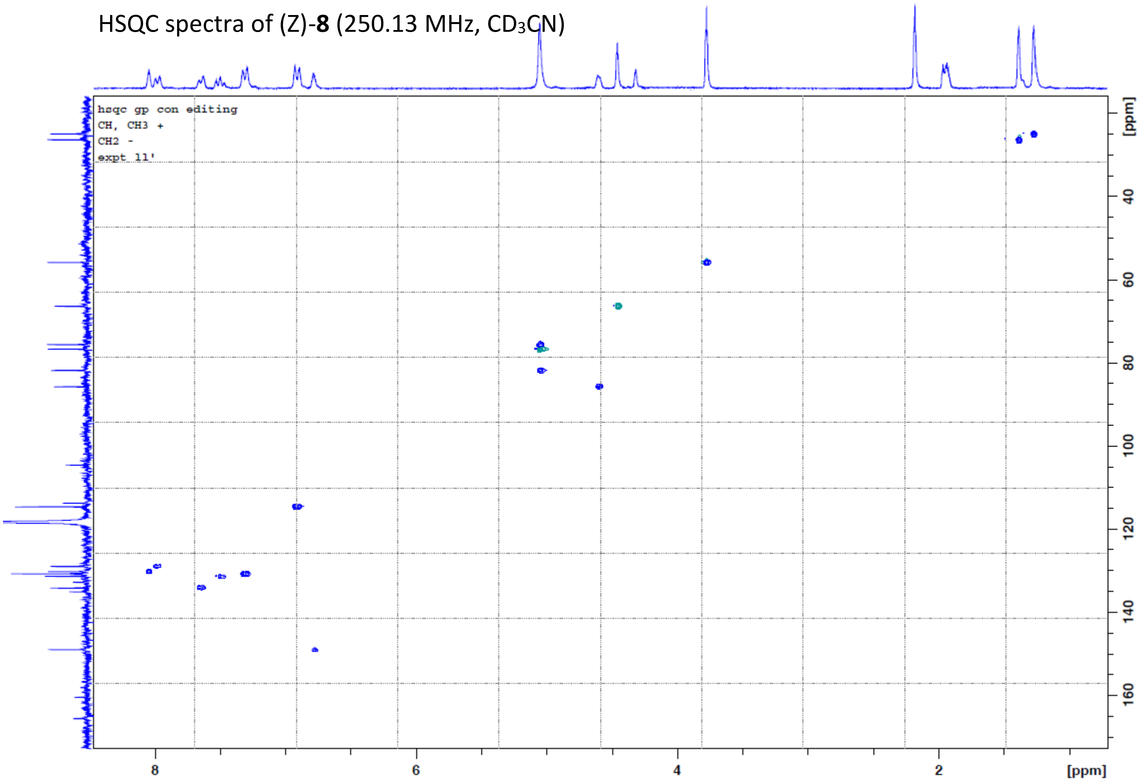

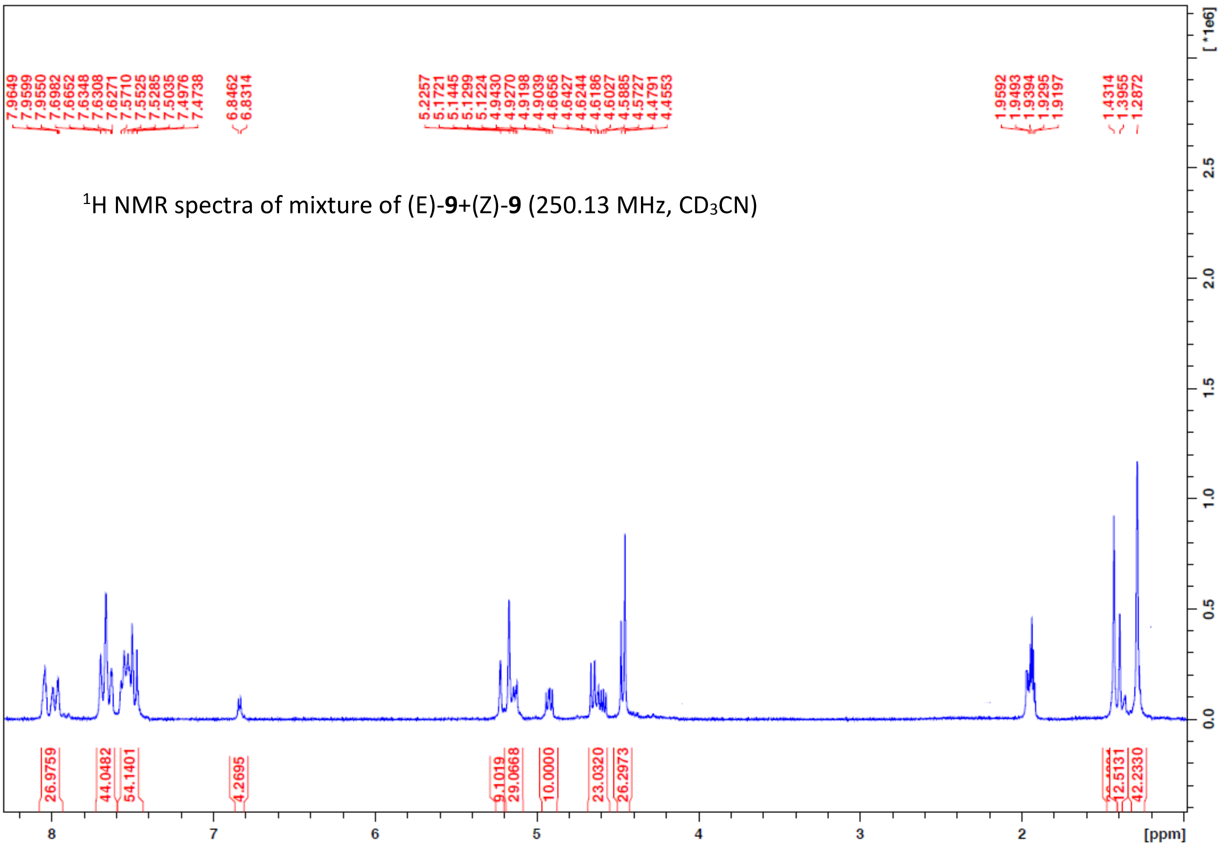

^{1}H NMR spectra of mixture of (E)-**9**+(Z)-**9** (250.13 MHz, CD_3CN)

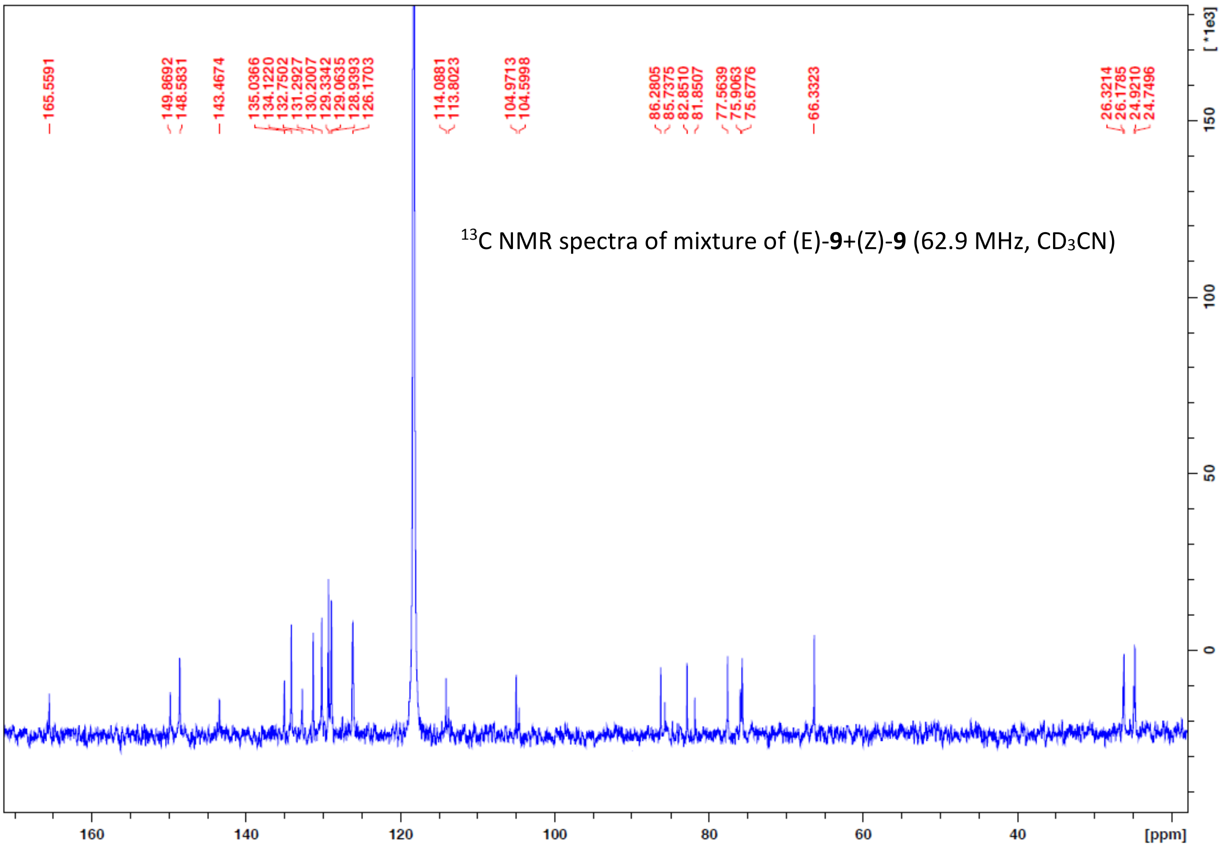

^{13}C NMR spectra of mixture of (E)-**9**+(Z)-**9** (62.9 MHz, CD_3CN)

COSY spectra of mixture of (E)-**9**+(Z)-**9** (250.13 MHz, CD_3CN)

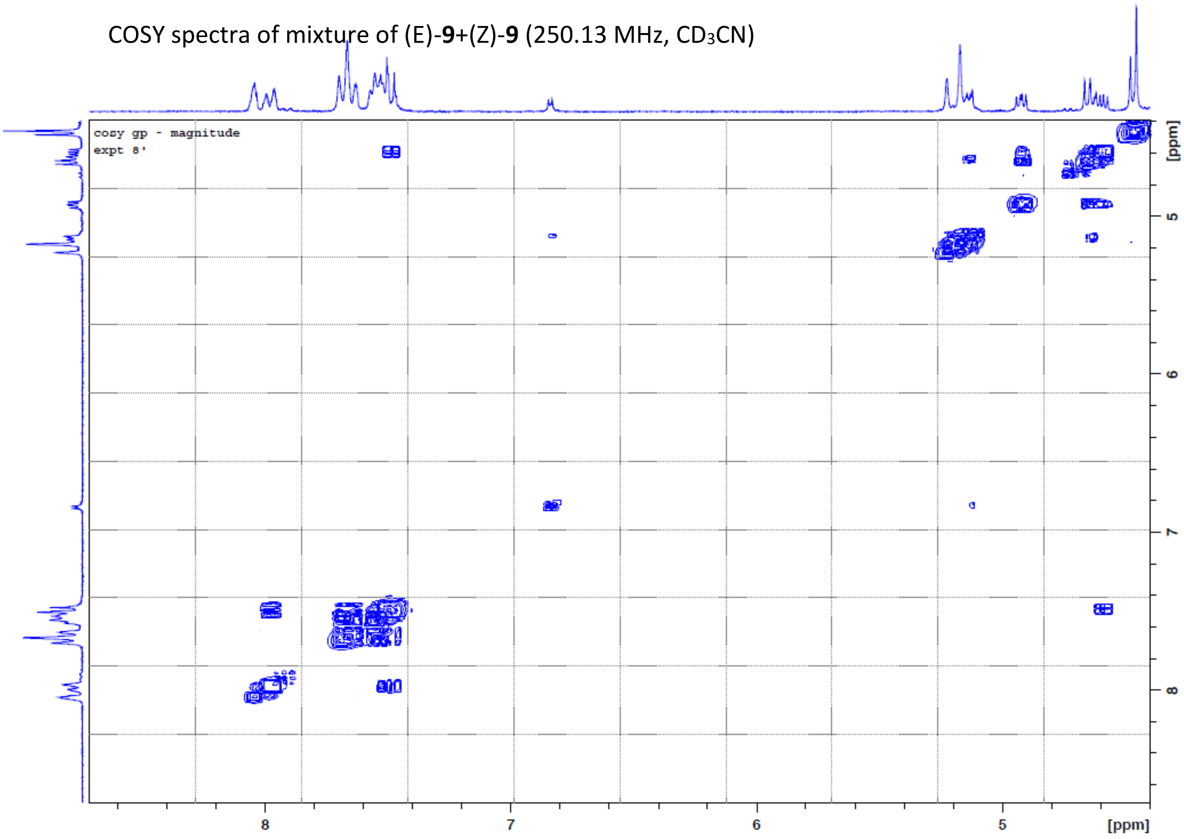

HSQC spectra of mixture of (E)-**9**+(Z)-**9** (400 MHz, CD_3CN)

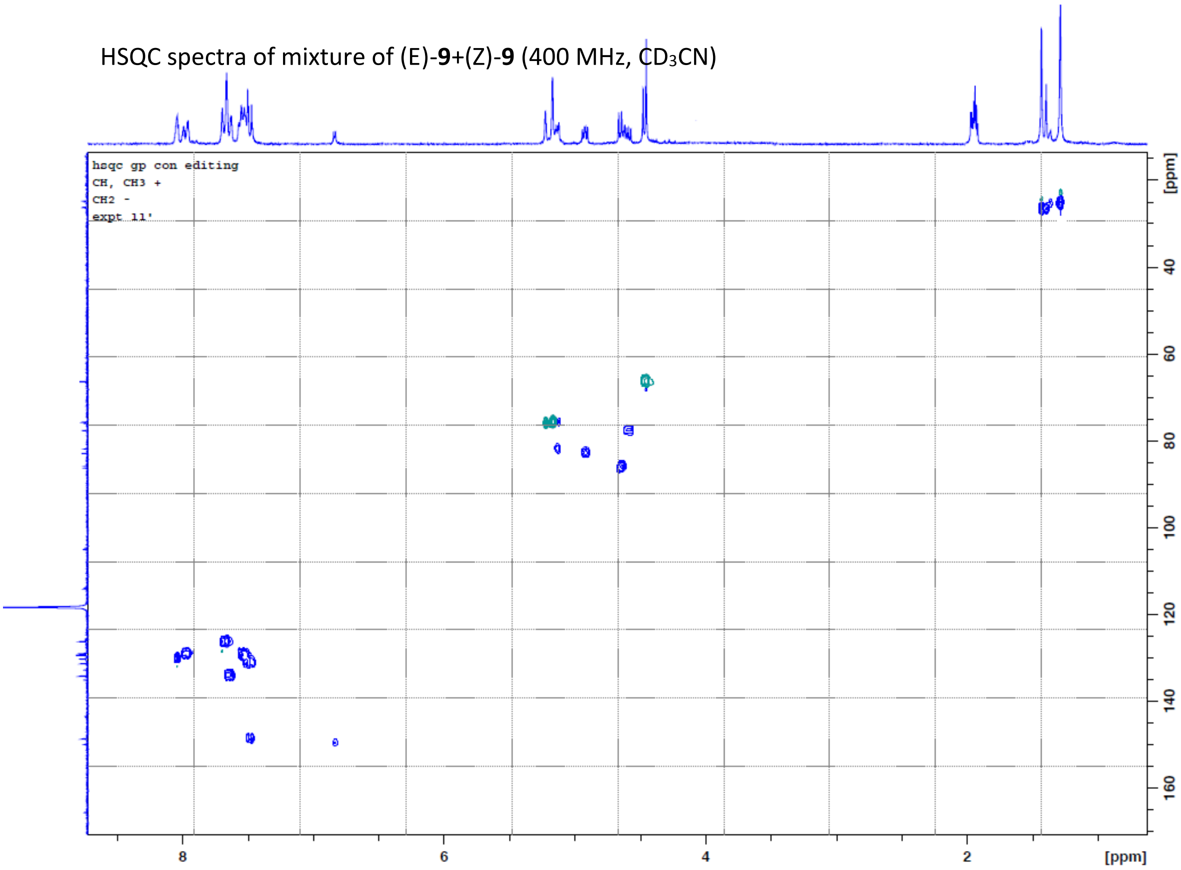

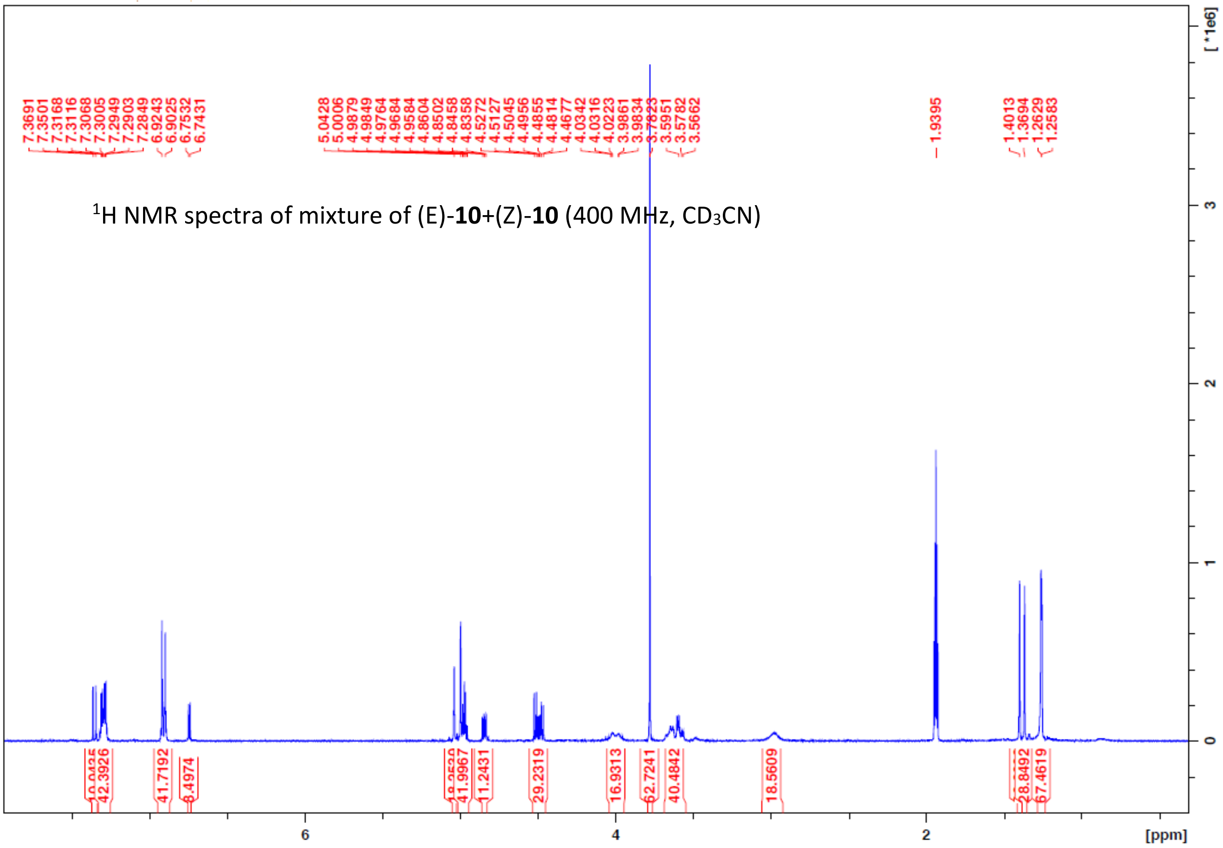

¹H NMR spectra of mixture of (E)-10+(Z)-10 (400 MHz, CD3CN)
7.3691
7.3501
7.3168
7.3116
7.3068
7.3005
7.2949
7.2903
7.2849
6.9243
6.9025
6.7532
6.7431
5.0428
5.0006
4.9879
4.9849
4.9764
4.9684
4.9584
4.8604
4.8502
4.8458
4.8358
4.5272
4.5127
4.5045
4.4956
4.4855
4.4814
4.4677
4.0342
4.0316
4.0223
3.9861
3.9834
3.5951
3.5782
3.5662
1.9395
1.4013
1.3694
1.2629
1.2583
42.3926
41.7192
8.4974
41.9967
11.2431
29.2319
16.9313
62.7241
40.4842
18.5609
28.8492
67.4619
6
4
2
[ppm]
[*1e6]
3
2
1
0

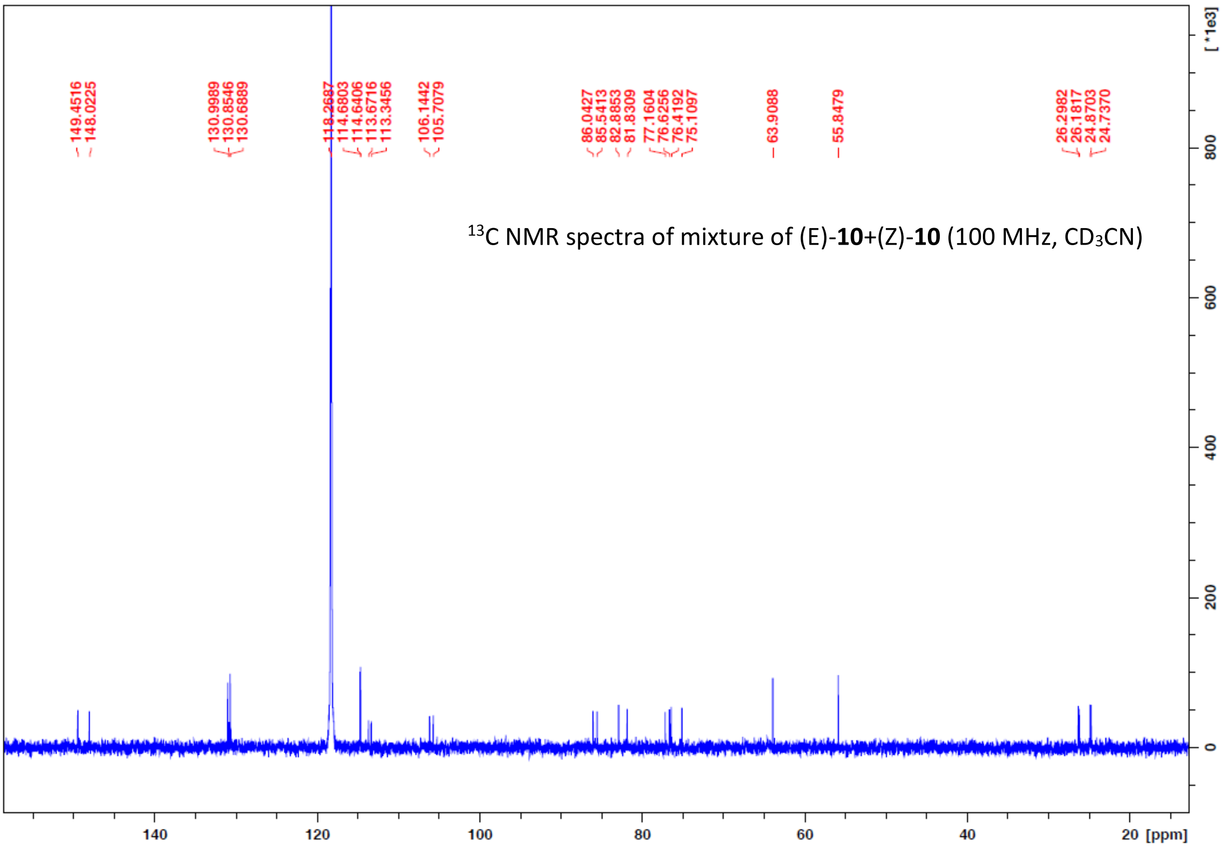

¹³C NMR spectra of mixture of (E)-10+(Z)-10 (100 MHz, CD3CN)
149.4516
148.0225
130.9989
130.8546
130.6889
114.6803
114.6406
113.6716
113.3456
106.1442
105.7079
86.0427
85.5413
82.8853
81.8309
77.1604
76.6256
76.4192
75.1097
63.9088
55.8479
26.2982
26.1817
24.8703
24.7370
140
120
100
80
60
40
20
[ppm]
[*1e3]
800
600
400
200
0

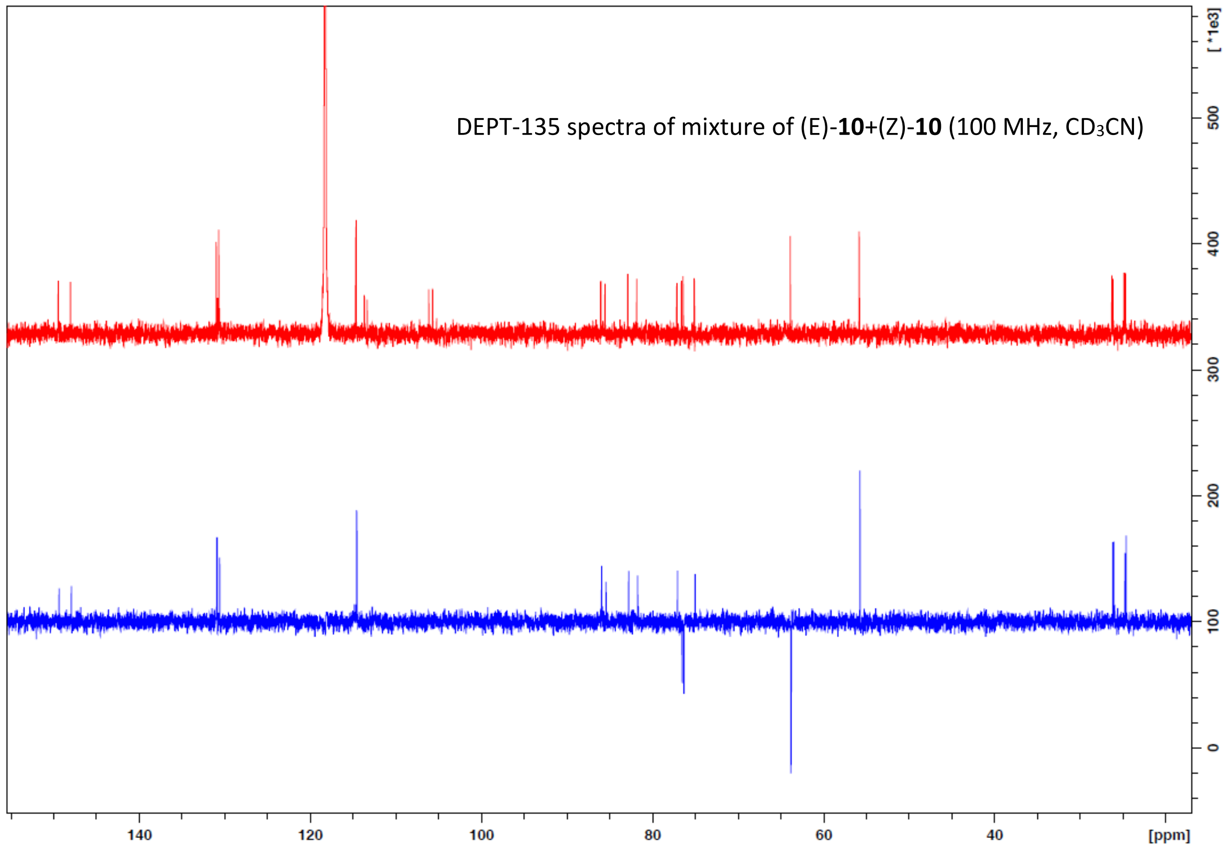
DEPT-135 spectra of mixture of (E)-10+(Z)-10 (100 MHz, CD3CN)
[*1e3]
500
400
300
200
100
0
140
120
100
80
60
40
[ppm]

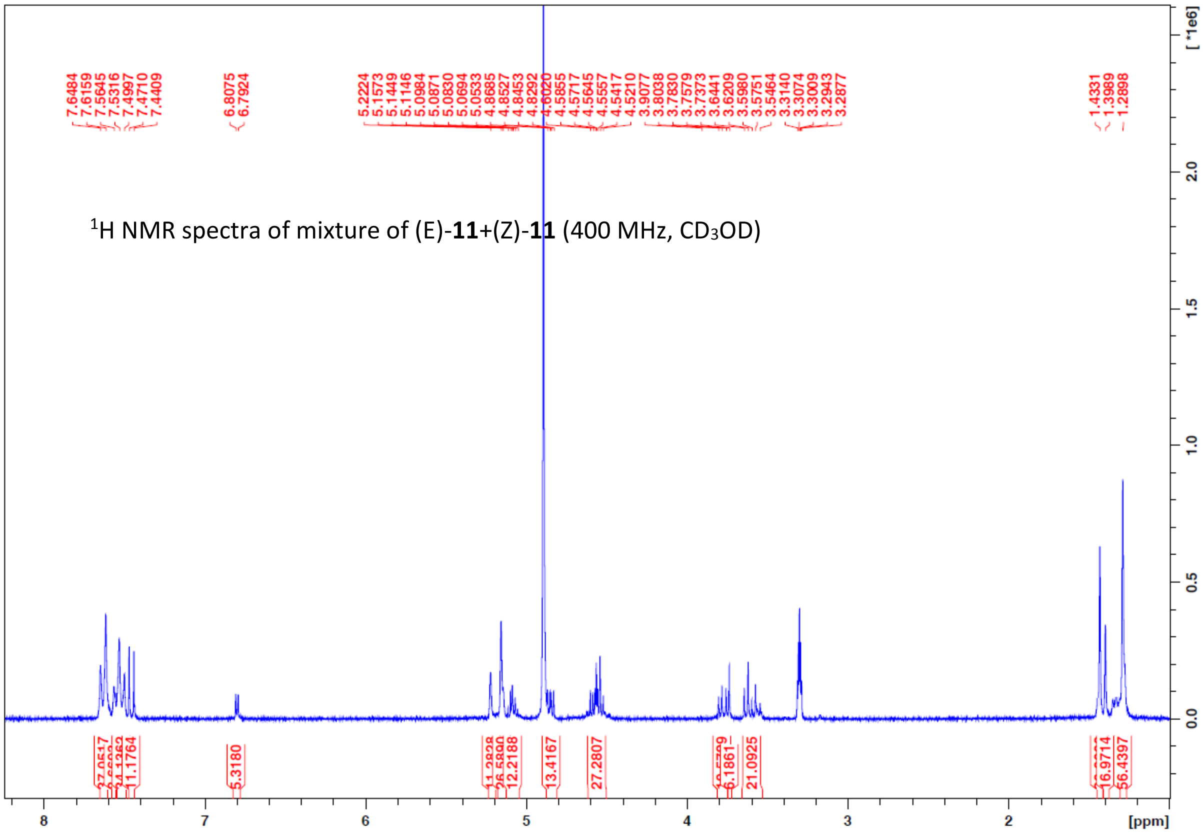
1H NMR spectra of mixture of (E)-11+(Z)-11 (400 MHz, CD3OD)
7.6484
7.6159
7.5645
7.5316
7.4997
7.4710
7.4409
6.8075
6.7924
5.2224
5.1573
5.1449
5.1146
5.0984
5.0871
5.0830
5.0694
5.0533
4.8685
4.8527
4.8453
4.8292
4.6020
4.5865
4.5717
4.5645
4.5557
4.5417
4.5210
3.9077
3.8038
3.7830
3.7579
3.7373
3.6441
3.6209
3.5980
3.5751
3.5464
3.3140
3.3074
3.3009
3.2943
3.2877
1.4331
1.3989
1.2898
[*1e6]
2.0
1.5
1.0
0.5
0.0
11.1764
5.3180
12.2188
13.4167
27.2807
6.1861
21.0925
16.9714
56.4397
8
7
6
5
4
3
2
[ppm]

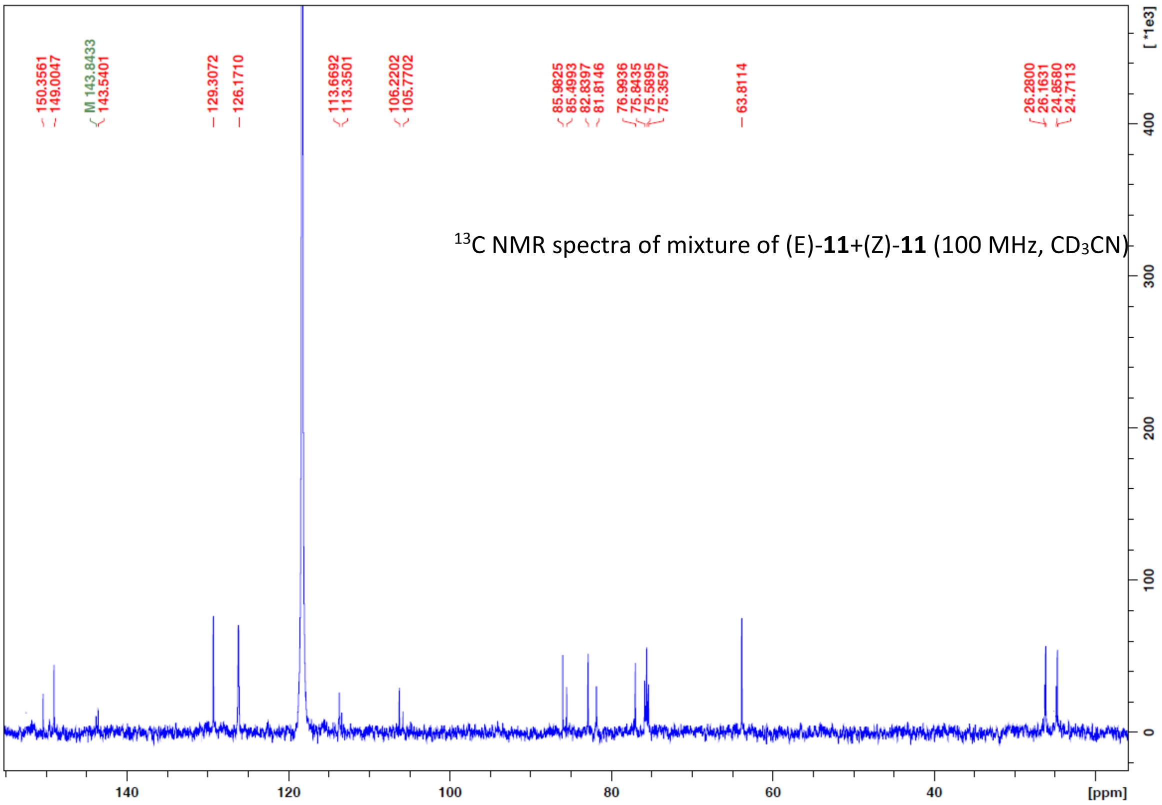

^{13}C NMR spectra of mixture of (E)-**11**+(Z)-**11** (100 MHz, CD_3CN)

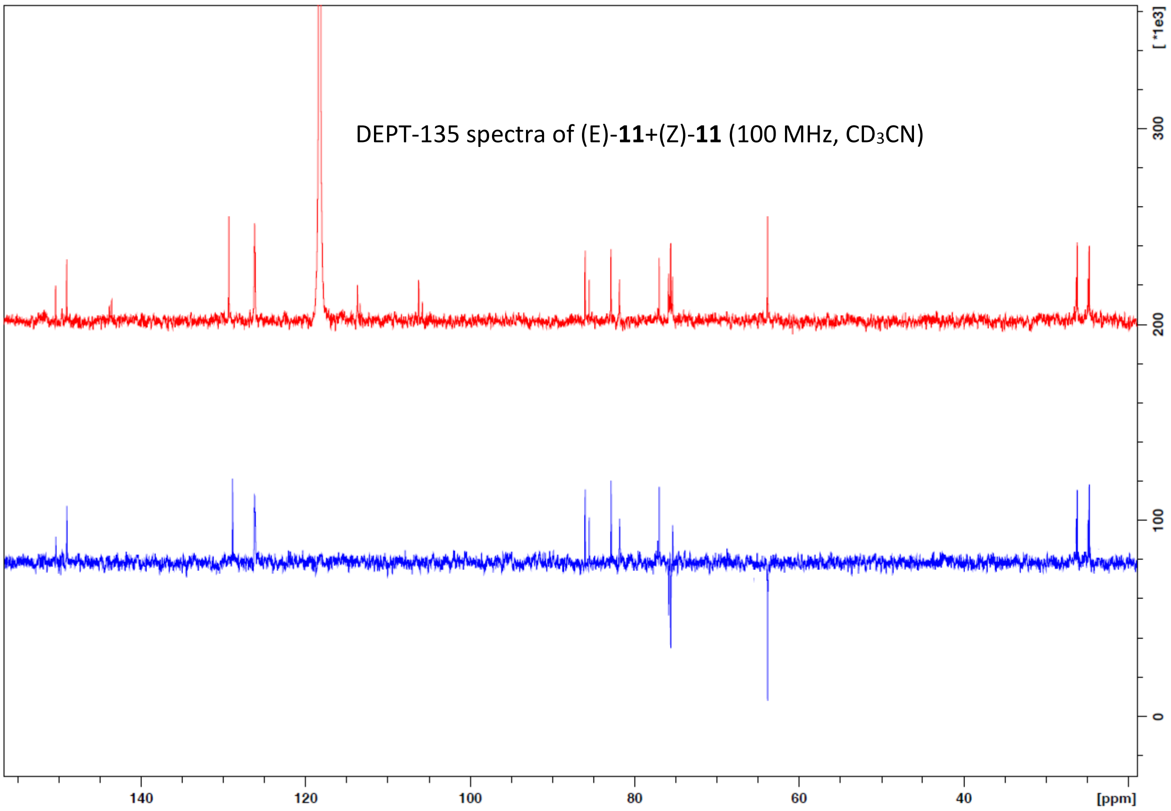

DEPT-135 spectra of (E)-**11**+(Z)-**11** (100 MHz, CD_3CN)

COSY spectra of mixture of (E)-**11**+(Z)-**11** (400 MHz, CD_3OD)

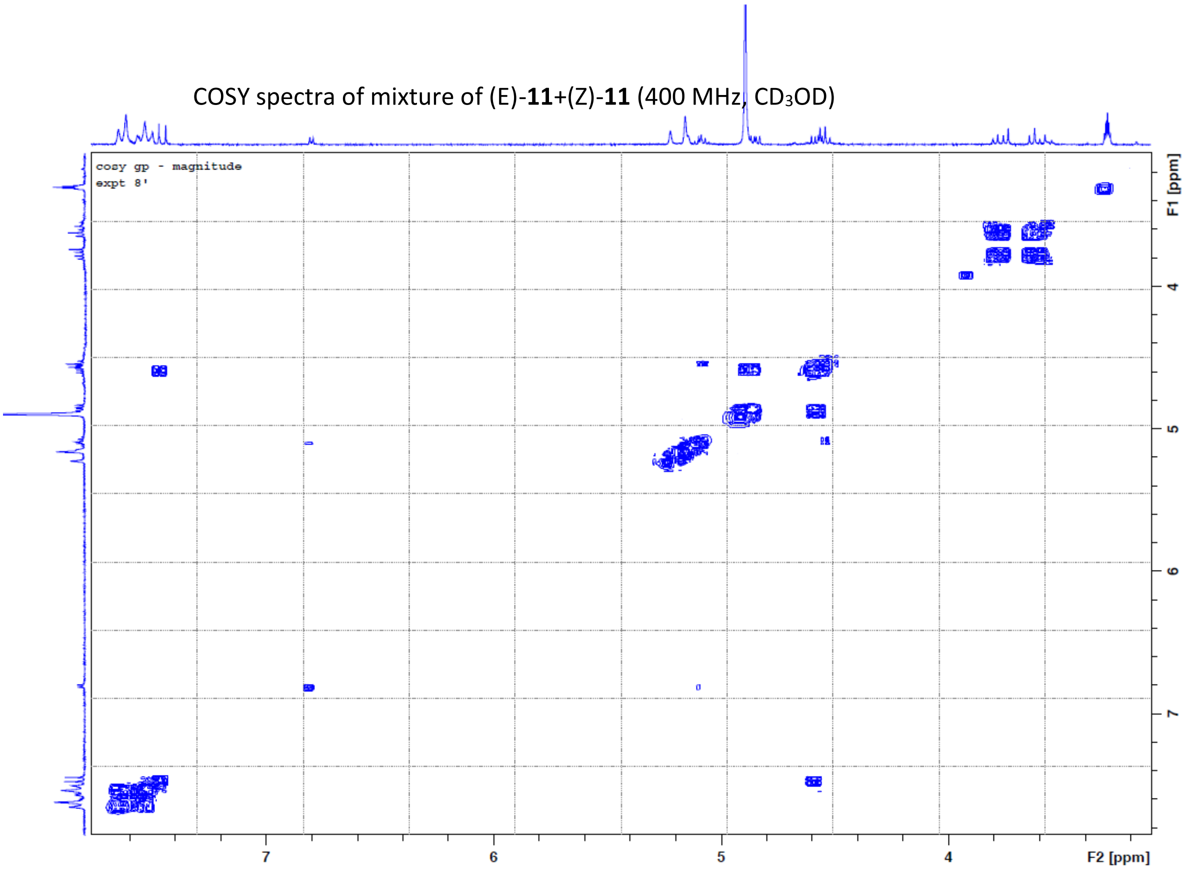

^{1}H NMR spectra of compound **12** (400 MHz, CD_3CN)

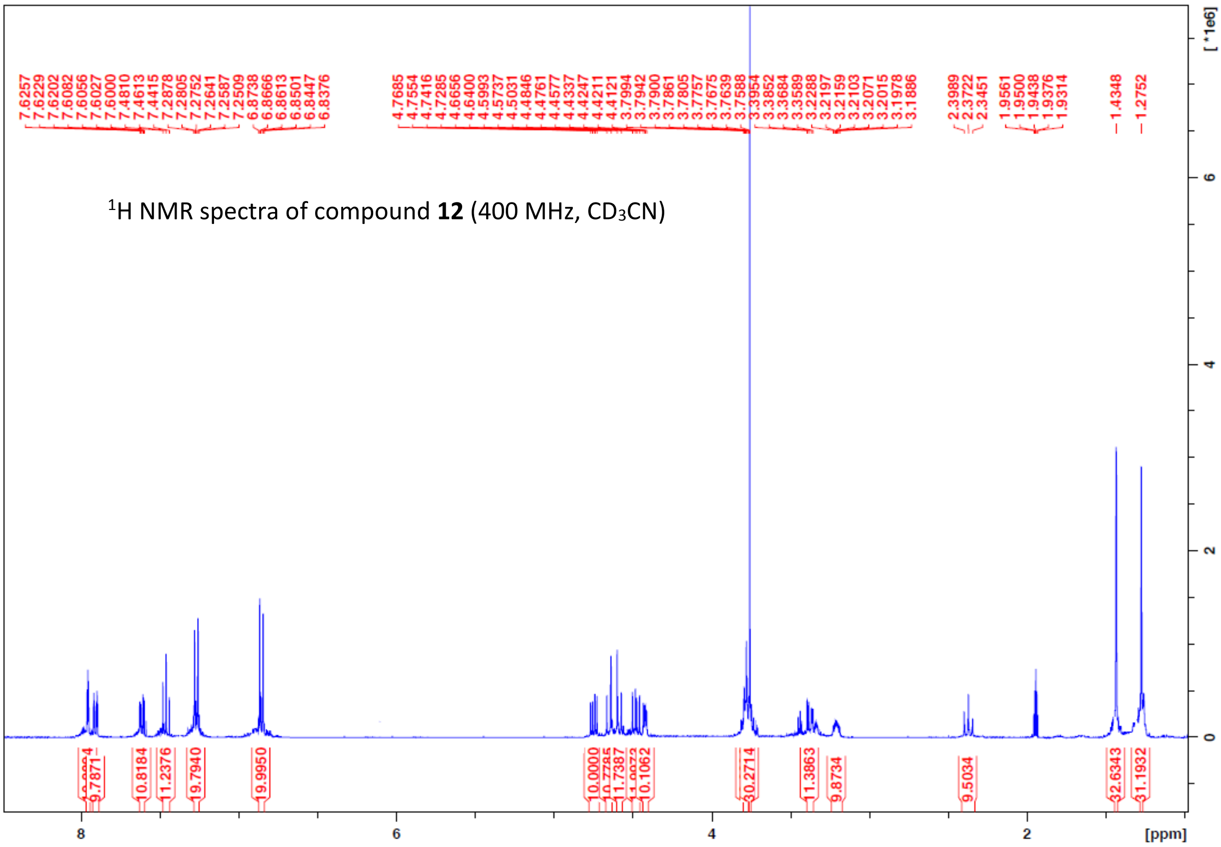

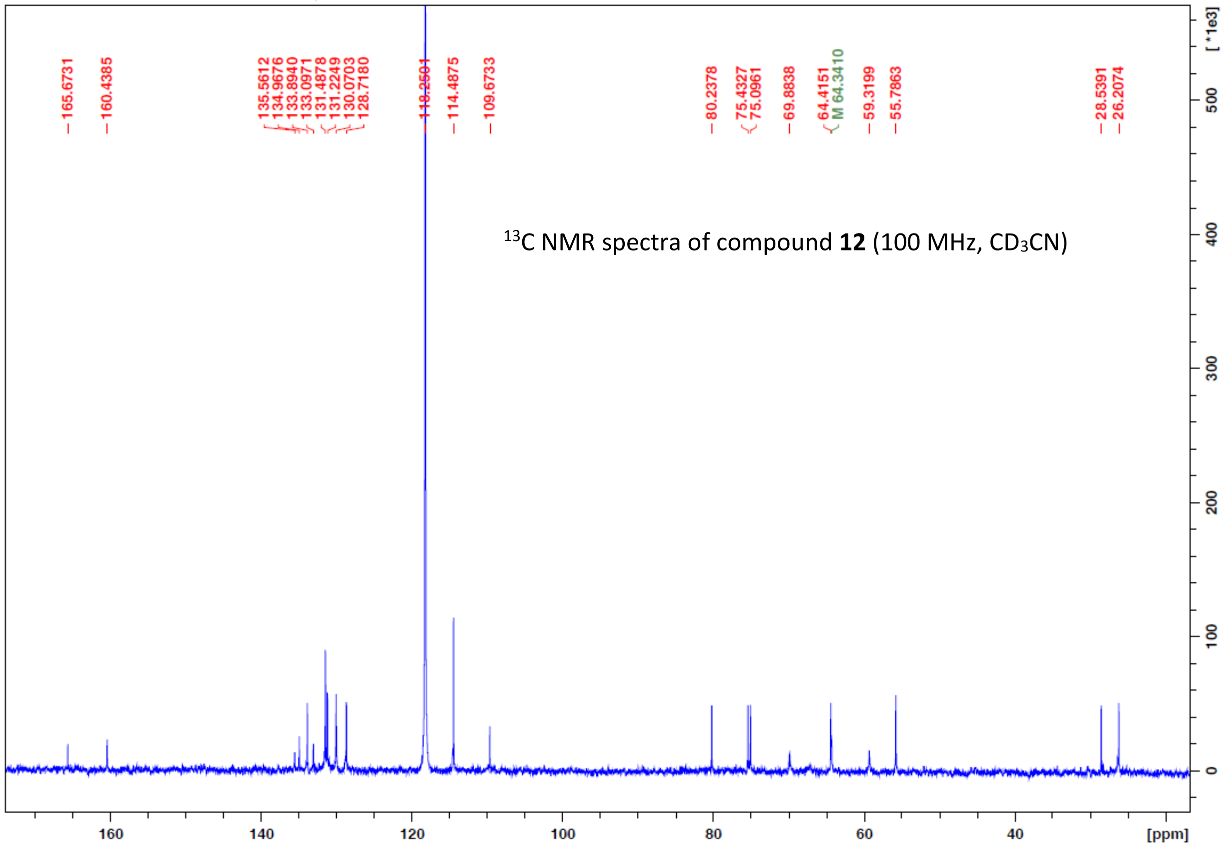

165.6731
160.4385
135.5612
134.9676
133.8940
133.0971
131.4878
131.2249
130.0703
128.7180
118.2501
114.4875
109.6733
80.2378
75.4327
75.0961
69.8838
64.4151
M 64.3410
59.3199
55.7863
28.5391
26.2074
^{13}C NMR spectra of compound **12** (100 MHz, CD_3CN)
[*1e3]
[ppm]

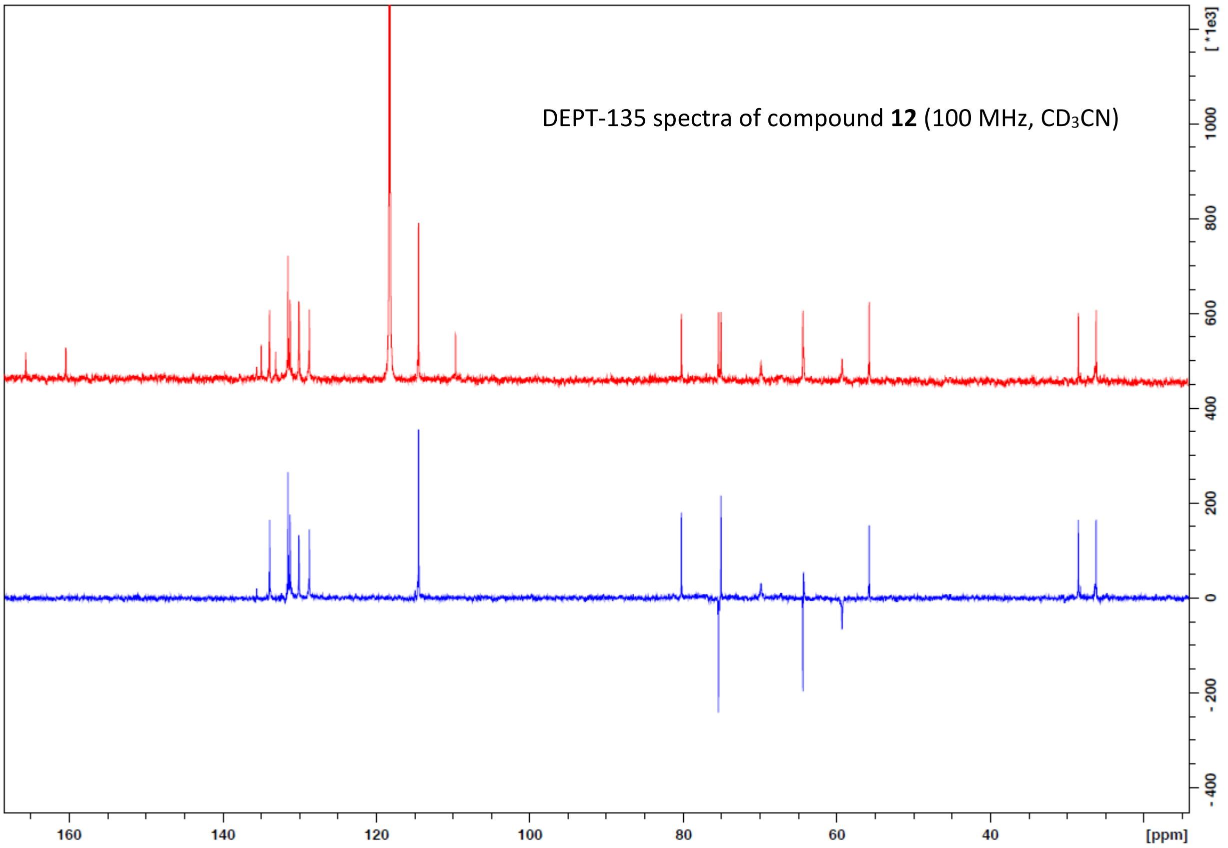

DEPT-135 spectra of compound **12** (100 MHz, CD_3CN)
[*1e3]
[ppm]

COSY spectra of compound **12** (400 MHz, CD_3CN)

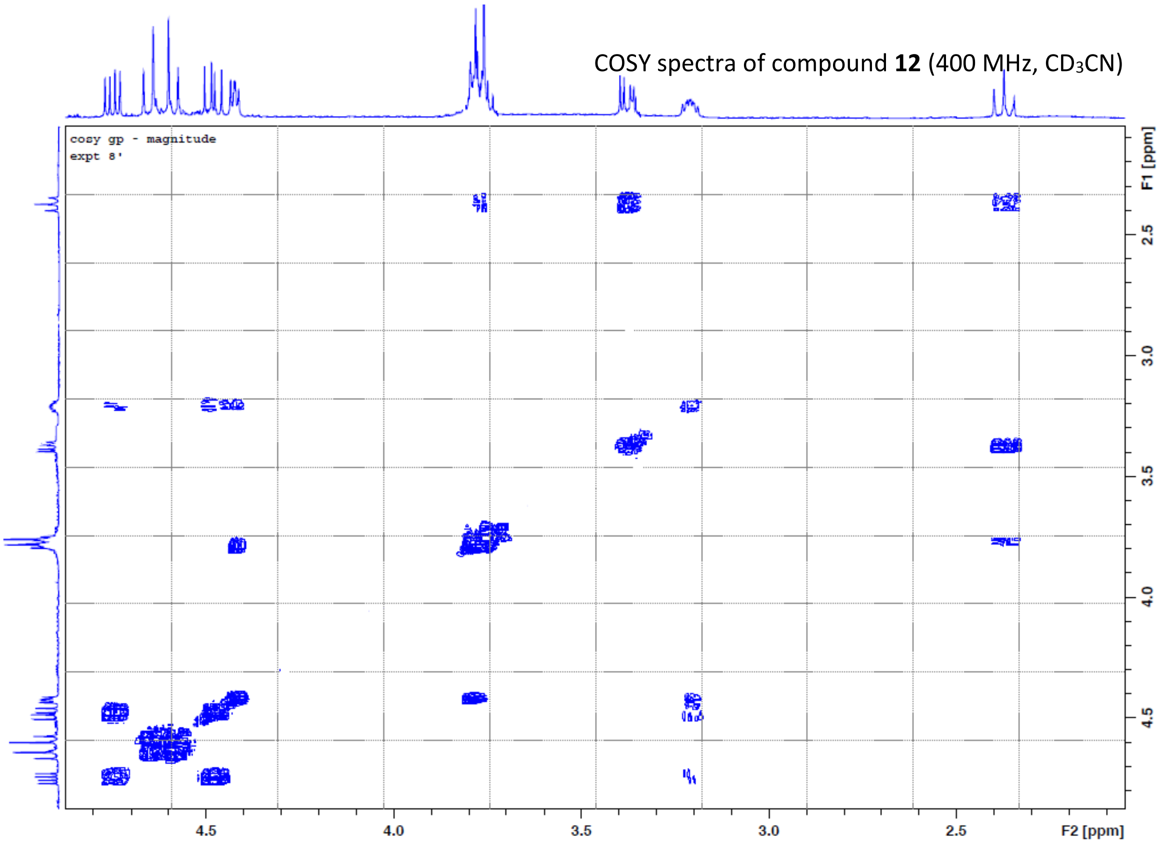

HSQC spectra of compound **12** (400 MHz, CD_3CN)

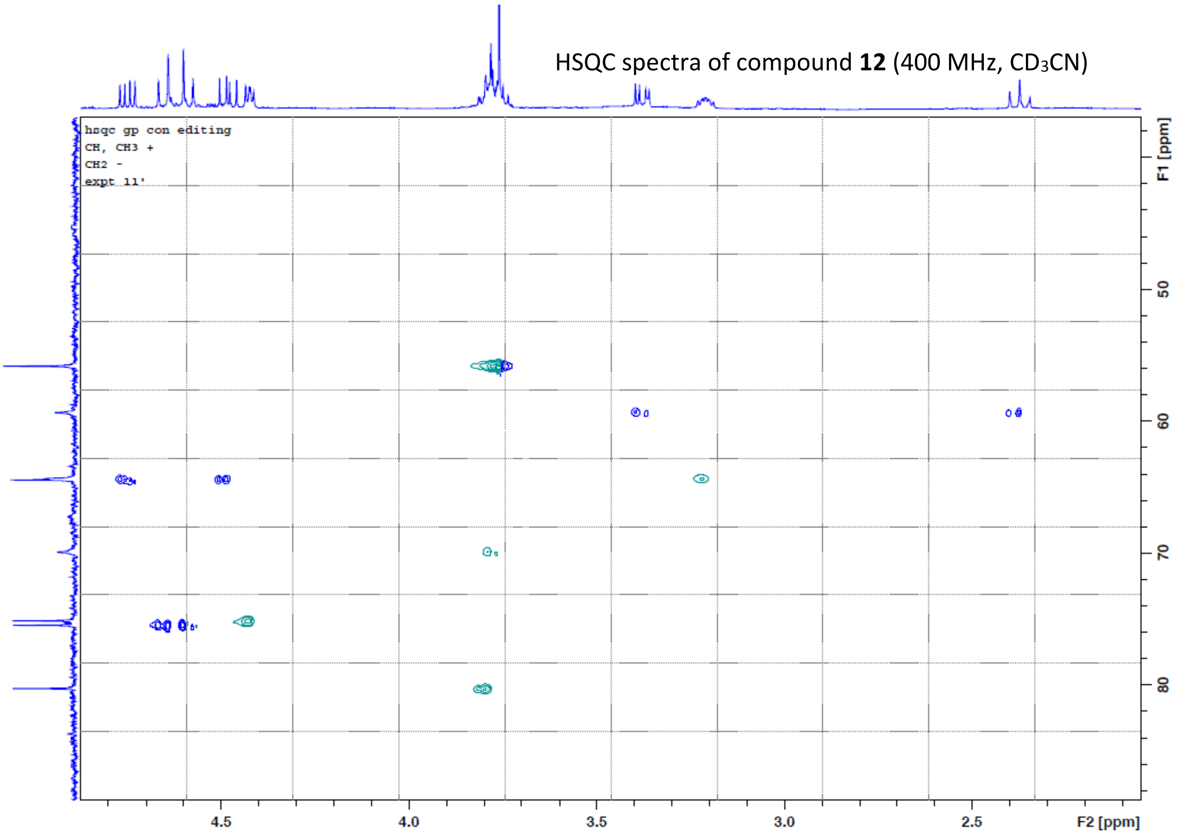

Supplement: Supplemental Material [file IENZ_A_1763331_SM5291.pdf]
